# Human cytomegalovirus at the maternal–fetal interface: an overview of pathogenesis, defence, and interventions

**DOI:** 10.1042/CS20250044

**Published:** 2026-06-25

**Authors:** Mahlaqua Noor, Catherine E. Aiken, Emma Poole, Naomi McGovern

**Affiliations:** 1 Department of Pathology, University of Cambridge, Cambridge, UK; 2 Loke Centre for Trophoblast Research, University of Cambridge, Cambridge, UK; 3 Department of Obstetrics and Gynaecology, NIHR Cambridge Biomedical Research Centre, University of Cambridge, Cambridge, U.K.

**Keywords:** Experimental models, Human Cytomegalovirus, Immune cells, Maternal-fetal interface

## Abstract

Human cytomegalovirus (HCMV) is the leading infectious cause of congenital disease worldwide and a major contributor to birth defects and neurodevelopmental disorders. Vertical transmission occurs when the virus breaches the maternal–fetal interface, a complex immunological environment where the placenta is in direct contact with either the uterine lining (decidua) or maternal blood covering the syncytium. The present review synthesises current understanding of HCMV pathogenesis at this critical interface, examining viral invasion mechanisms, host immune responses, and immune evasion strategies. We outline the structural and cellular features of the placenta and decidua, highlighting key immune populations such as uterine natural killer cells and their roles in antiviral defence. The present review examines the mechanisms of HCMV dissemination across the placenta alongside placental-specific defences to infection and details on how HCMV subverts both innate and adaptive immunity to establish and maintain infection. Critical gaps in diagnostics, the limited efficacy of current antiviral therapies, and obstacles in vaccine development are reviewed, underscoring the urgent need for prevention strategies. Finally, we assess experimental models, placental explants, primary trophoblast and decidual organoids, and microfluidic placenta-on-chip systems that are advancing insights into cell-type-specific tropism, immune interactions, and mechanisms of transplacental HCMV transmission. Continued research into host–virus interactions at the maternal–fetal interface and improved model systems are critical for developing targeted prevention and treatment strategies to reduce the burden of congenital HCMV infection.

## Introduction

Congenital human cytomegalovirus (HCMV) infection begins when the virus breaches the placental barrier. The placenta, the first organ formed by the embryo, mediates nutrient and gas exchanges between mother and fetus. The decidua, a transformed maternal endometrium, supports placental function and harbours immune cells that balance surveillance and aid in implantation. There are two main anatomical sites where placental trophoblast cells are in direct contact with maternal cells. The invading extravillous trophoblast (EVT) cells move into the maternal decidua early in gestation and interact with tissue immune cells. Once the placental villi are formed, maternal blood enters the intervillous space and is in contact with the syncytiotrophoblast (STB) covering the villous tree. Recent advances have greatly expanded our understanding of this interface, yet critical gaps remain. In the present review, we synthesise current knowledge and highlight unresolved questions, with a particular focus on experimental models used to study placental infection and unique challenges posed by HCMV, the leading cause of congenital infection worldwide.

## Historical milestones in understanding human cytomegalovirus

HCMV was first recognised as a cause of congenital disease in the 1950s, although its pathological effects had been noted decades earlier. In 1881, Ribbert described inclusion-bearing cells in infant tissues, mistakenly identifying these enlarged cells as protozoa [1]. Later in 1921, Goodpasture and Talbert reported that the cells resembled those seen in varicella infections in skin and were likely the result of viral activity, coining the term *cytomegalia infantum* [2]. A major breakthrough came in 1956 when three independent research groups, Smith in St. Louis, Rowe and colleagues in Bethesda, and Weller in Boston, simultaneously isolated human CMV strains [3–5]. In 1960, Weller and colleagues named the virus ‘cytomegalovirus’ after isolating it from the urine of infants with generalised disease [6,7].

The development of cell culture techniques in the mid 20th century enabled CMV propagation, although detection in clinical samples required prolonged cultivation periods of several weeks. Initial vaccine studies using live attenuated CMV were conducted in the mid-1970s, including the AD169 vaccine developed by Elek and Stern in 1974 [8] and the Towne 125 vaccine created by Stanley Plotkin and colleagues in 1975 [9]. Another significant advance came in the 1980s with the introduction of rapid diagnostic methods combining centrifugation-enhanced inoculation with CMV antigen detection, followed by direct molecular detection, reducing turnaround times to clinically actionable intervals [10]. In 1989, the FDA approved ganciclovir, marking the first effective antiviral therapy against HCMV [11], and by 1990, its use for congenital CMV pneumonia was reported [12]. The publication of the first complete CMV genome sequence in 1990 [13] marked another milestone in understanding viral biology, followed by the sequencing of the Merlin strain in 2004, which established the reference genome for wild type HCMV [14]. In 2005, oral ganciclovir was first reported as a treatment for congenital CMV in pregnancy [15]. Around the same time, major molecular advances reshaped understanding of HCMV entry mechanisms. In particular, the discovery in 2005 [16] of the pentameric complex, comprising gH, gL, UL128, UL130, and UL131A, revealed that these five proteins assemble into a specialised envelope glycoprotein complex that is essential for HCMV infection of epithelial, endothelial, and myeloid cells. By defining the structural basis of this broader tropism, the pentameric complex has emerged as a major target for HCMV vaccine development.

**Figure 1:**
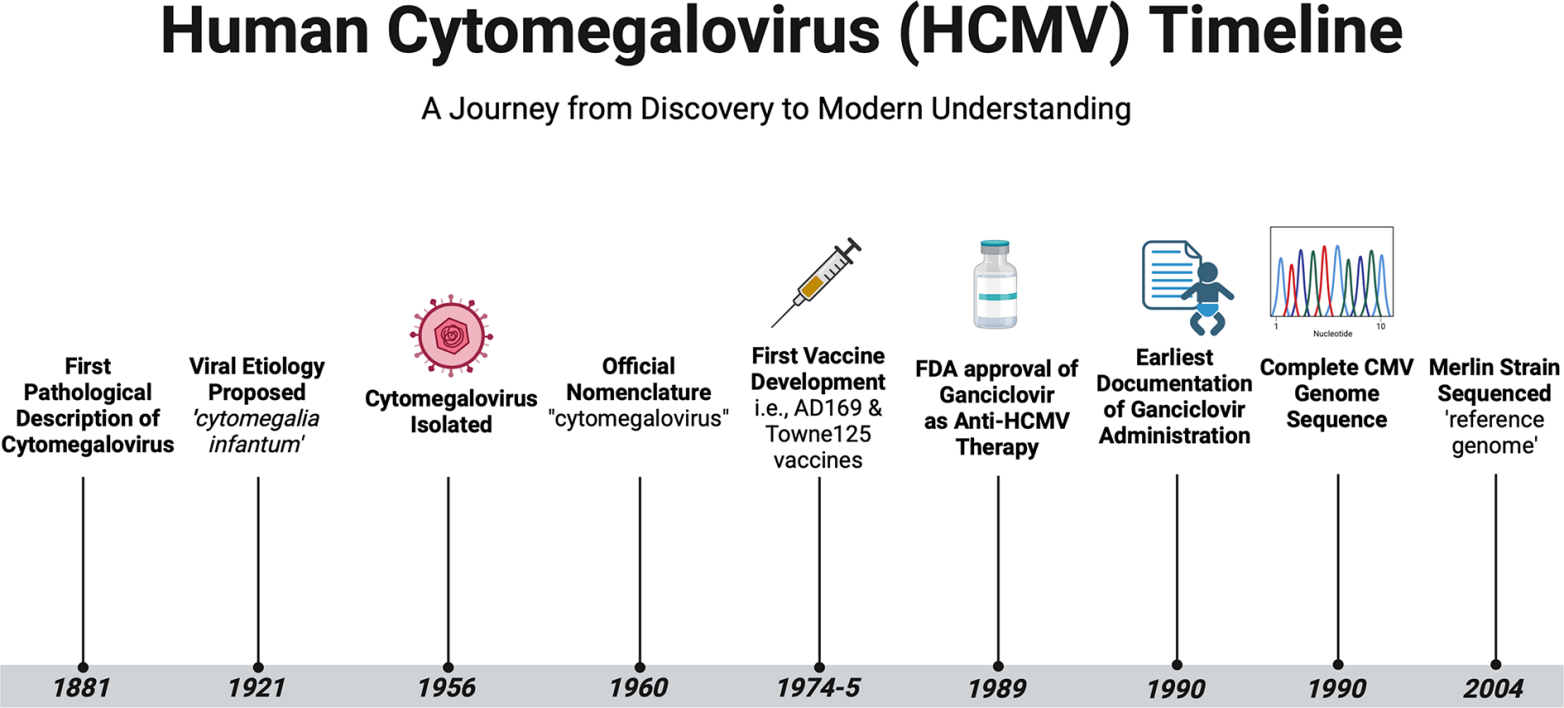
Timeline of major discoveries in the field of HCMV with a focus on congenital CMV Made in Biorender.

Collectively, these achievements established the scientific framework for elucidating CMV pathogenesis and continue to inform contemporary strategies for preventing and treating congenital infection (Figure 1).

## The disease burden of congenital HCMV

HCMV, a member of the *Betaherpesviriniae* subfamily, establishes lifelong latency following primary infection and can reactivate later in life. It is a linear double-stranded DNA virus with an exceptionally large genome of approximately 236 kb, the largest among human-infecting herpes viruses. This genome encodes up to ∼750 open reading frames (ORFs) and at least 26 mature microRNA genes distributed throughout the viral genome [17,18]. Many of these genes are dedicated to immune evasion, maintenance of latency, and broad cellular tropism, enabling HCMV to infect diverse cell types [19].

HCMV can be acquired through natural routes, including both horizontal and vertical transmission. Horizontal person-to-person spread occurs through contact with infected bodily fluids like saliva, urine, blood, and semen [20]. Vertical transmission may occur transplacentally during gestation or transvaginally during delivery, and there is also evidence supporting transmission through breastmilk [21]. Following primary infection, the virus establishes a latent reservoir that can reactivate (nonprimary infection/reactivation), or the maternal host may become infected with a different strain (nonprimary/reinfection) [22].

The risk of mother-to-child transmission is highest in the third trimester, with an estimated 70% risk if the mother experiences a primary infection compared with 30% risk in the first trimester [23,24]. However, the mechanisms underlying this trimester-dependent variation in vertical transmission risk remain incompletely understood. Higher transmission rates in the third trimester likely reflect increased maternal viraemia during primary infection and structural maturation of the placental villous architecture, notably the formation of vasculosyncytial membranes, which thin the STB layer and bring fetal capillary endothelium into proximity with the maternal circulation, potentially favouring haematogenous viral transmission. The comparatively lower transmission risk reported in the first trimester may partly reflect survivorship bias, as early gestational infection may disproportionately result in miscarriage, meaning affected pregnancies are underrepresented in transmission estimates. Changes in immune cell properties across gestational stages may also contribute to this difference, but this remains poorly understood [25]. Primary maternal infection carries the greatest risk for vertical transmission and severe congenital disease due to the absence of pre-existing immunity [26–29].

In addition to the placenta and fetus, infection of the chorionic membranes with HCMV has also been described [30]. This may account for the high rates of preterm birth in CMV-affected pregnancies by predisposing to premature rupture of the membranes. More research is needed to understand whether transmission can occur across the chorionic membranes. Globally, HCMV is the leading cause of congenital infection and the most common infectious contributor to birth defects. In high-income countries, it affects over 1 in 200 live births, while the prevalence is approximately three times greater in low- and middle-income countries [31,32]. Congenital CMV infection can result in lifelong complications, including birth defects, such as sensorineural hearing loss, microcephaly, and neurodevelopmental disabilities. Approximately 10% of HCMV-positive infants exhibit neurological impairment from birth [33,34].

## The maternal–fetal interface

To infect the fetus, HCMV must traverse the placental barrier at the maternal–fetal interface.

## The placenta

**Figure 2:**
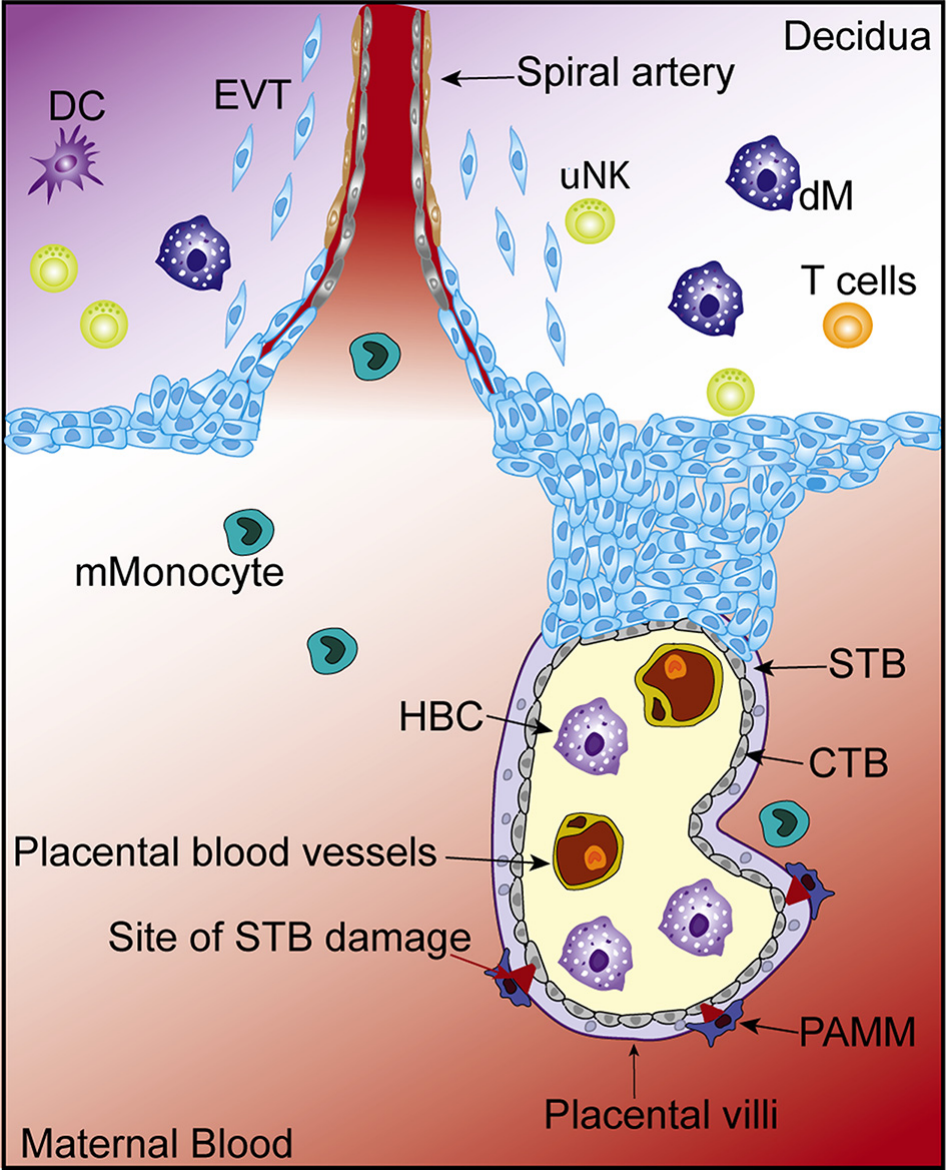
The maternal–fetal interface at 12 weeks gestational age Hofbauer cells (HBCs) are found within placental villi. The outer layer of the placental villi is covered by the STB. Placental-associated maternal macrophages (PAMM) adhere to sites of damage on the STB. CTBs are found under the STB as a continuous layer and become progressively less abundant by term. Where villi contact decidua (anchoring villi), the inner CTB layer differentiates into EVT, which migrates into the decidua toward the spiral arteries, which are transformed to increase maternal blood flow to the intervillous space. The immune cells present in the decidua include uterine natural killer (uNK) cells, decidual macrophages (dM), dendritic cells (DCs), and T cells.

In humans, placental formation begins at 5 days post conception when the blastocyst implants into the maternal endometrium [35]. Trophoblast cells arise from the trophectoderm, which forms the outer layer of the blastocyst and differentiates into two main lineages. Cytotrophoblast (CTB) cells proliferate rapidly to form branching villi covered by multinucleated STB that are in contact with maternal blood, the major site of nutrient, gas exchange, and transport. EVTs emerge out of anchoring villi and, upon contacting the decidua, migrate into maternal tissue [36–39] (Figure 2).

EVTs play a critical role in remodelling the media of the maternal spiral arteries, which increases maternal blood to the intervillous space from 8 to 10 weeks of gestation. The efficiency of this process profoundly influences placental development and function; impaired blood flow can damage the villous tree and lead to fetal growth restriction. As maternal blood flow to the placenta increases with increasing gestation, so too does the risk of fetal exposure to any maternal circulating viral load [36–38].

At 18 days post-conception, placental macrophages, termed HBCs, are found within the placental villous stroma [40]. Around 8 weeks post-conception, fetal blood vessels within the placenta mature [41], initiating chorionic circulation [42]. Alongside these anatomical changes, the dynamics of placental transport also shift, including a rise in oxygen tension and the onset of maternal IgG transfer to the fetus from the second trimester onwards [43,44]. Our previous work has shown that the properties of HBCs evolve across gestation, reflecting adaptations to local signalling cues [45]. These changes are clinically relevant; by term, HBCs exhibit reduced antimicrobial activity against the transplacental pathogen *Listeria monocytogenes*, compared with the first trimester [45].

Whether other placental cell types undergo similar functional changes across gestation remains unclear. While certain populations, such as CTBs, decline in abundance by term, the extent to which their functional and antimicrobial properties vary throughout gestation remains poorly understood.

## Immune cell populations in the decidua

uNK cells are the predominant maternal immune population in the secretory phase of the decidualising human endometrium prior to the initiation of pregnancy. Following blastocyst implantation, the decidualisation continues with uNK cells accounting for approximately 70% of placental leukocytes during the first trimester [46]. Their numbers decline progressively toward term [47,48]. Phenotypically, uNK cells are characterised as CD56^bright^CD16^neg^, in contrast with 90% of peripheral blood NK cells which are CD56^dim^CD16^pos^. There are three subpopulations of uNK cells: uNK1, uNK2, and uNK3 [46,49–51]. Distinguishing markers include CD39 and LILRB1 (uNK1), ITGB2 and ANA1 (uNK2), and TIGIT (uNK3). Optimal uNK cell function is essential for successful pregnancy with a key role in regulating EVT invasion [49,50,52]. Detailed mechanisms underlying how these regulate a successful pregnancy are reviewed in depth elsewhere [53].

Although less abundant than uNK in early pregnancy, the decidua also harbours mononuclear phagocytes including DCs and macrophages. Macrophages in nonpregnant endometrial tissue have been comprehensively reviewed [54]. Two major decidual macrophage subsets were initially described as CD11c^hi^ and CD11c^lo^ cells [55], later refined by single-cell transcriptomic studies as dM1 and dM2 [49]. Further analyses integrating localisation and transcriptomic data introduced an additional classification: decidua basalis-associated macrophages (decBAMs) and decidua parietalis-associated macrophages (decPAMs). Comparative transcriptomics revealed that decBAMs correspond to dM1, whereas decPAMs align with dM2 profiles identified by scRNA-seq [49].

Phenotypically, decBAMs and decPAMs can be distinguished by their respective expression patterns: CD11c^+^HLA-DR^lo^ (decBAMs) and CD11c^lo^ HLA-DR^hi^ (decPAMs). DecBAMs exhibit proliferative capacity, and evidence suggests decPAMs may differentiate into decBAMs upon exposure to EVT-derived signalling cues. Functionally, *in vitro* studies indicate decBAMs promote regulatory T cell (Treg) induction, whereas decPAMs, consistent with their high HLA-DR expression, could potentially drive T cell activation [56].

Due to their relative recent characterisation, these subpopulations have not yet been studied in the context of infection. However, decPAMs express the Zika virus entry receptor *AXL*, suggesting a potential role in the transmission of this virus [57].

An additional maternal immune population, which we described as PAMM, also referred to as macrophage 3, has previously been described [49,58]. Although these macrophages are sparse, they adhere to sites of STB damage [59,60]. Their transcriptional profile is enriched for scar-associated macrophages found in human cirrhotic livers, and they express factors, such as *MMP-9* and *fibronectin*, suggesting a role in STB repair [61]. PAMMs exhibit low but detectable CCR2 expression, indicative of their monocytic origin. Importantly, their positioning at sites of damaged STB could also facilitate transplacental infection [60], providing a potential route for pathogens such as HCMV via adherence of infected monocytes.

DCs are professional antigen-presenting cells that initiate and shape T cell responses. DCs compromise several specialised subsets, each equipped to detect and respond to diverse pathogens and environmental cues. These include plasmacytoid DCs (pDCs), XCR1^+^ type 1 conventional DCs (DC1s), CD172a^+^ type 2 conventional DCs (DC2s), recently subdivided into DC2A and DC2B, and CD16/32^+^CD172a^+^ DC3s, which exhibit monocyte-like characteristics [62,63]. Whether all these subsets are found in the human decidua or their specific roles remain unclear. uNK cells secrete high quantities of XCL1, an important molecule for the regulation of EVTs and a potent chemoattractant for DC1 [49,52,64]. Consequently, DC1s appear relatively enriched in the decidua compared with the other DC subsets [49]. The identity of DC2-like cells detected by single-cell transcriptomics is uncertain, as they might represent blood contaminants [49]. While several studies have characterised decidual DCs [65], many predate and do not align with the current nomenclature for DC subsets [66]. Thus, the composition and functional potential of decidual DC subsets remain to be fully elucidated. Furthermore, the lack of lymphatic vessels, as documented earlier [67], but still inadequately investigated, may restrict T cell priming against trophoblast antigens by DCs.

Early in pregnancies, the decidual T cell compartment comprises approximately 10%–20% CD3^+^ TCRαβ^+^ T cells, of which 30%–45% are CD4^+^ T cells and 45%–75% are CD8^+^ T cells [68,69]. Decidual T cell biology has been comprehensively reviewed elsewhere [68,70]. Briefly, evidence indicates that effector memory T cells adapt during pregnancy by modulating their function, proliferation, and migratory capacity to promote immune tolerance at the maternal–fetal interface [68,70–73]. For example, expression of key immune checkpoint proteins, PD-1, Tim-3, CTLA-4, and LAG-3, is markedly increased on decidual effector memory T cells [73]. Decidual CD8^+^ effector cells, collected from decidual samples in term pregnancy, exhibit reduced expression of cytolytic molecules such as perforin and granzyme B compared with their peripheral blood counterparts [74], suggesting that the decidual microenvironment dampens CD8^+^ effector activity to maintain tolerance to fetal antigens. However, this suppression is not irreversible: upon *in vitro* stimulation, decidual CD8^+^ T cells degranulate, proliferate, and produce IFN-γ, TNF-α, perforin, and granzymes, demonstrating their retained capacity to respond to proinflammatory stimuli, including infection [75].

## HCMV at the maternal–fetal interface

Exactly how HCMV crosses the placental barrier is incompletely understood, largely due to the virus's strict host range and poorly defined entry routes. The most likely route, supported by anatomical and epidemiological evidence, is that the virus crosses through the trophoblast covering the villi from maternal blood. Maternal viraemia provides direct exposure of the maternal–fetal interface to circulating virus, and epidemiological data consistently show that higher maternal viral burdens are associated with increased risk of fetal infection. Consequently, transplacental spread during episodes of viraemia is considered the most plausible mechanism of vertical transmission [76,77].

A maternal viraemic episode can arise from primary infection, reactivation, or reinfection. The virus may disseminate systemically freely or via infected leukocytes such as monocytes, DCs, and CD34^+^ hematopoietic progenitor cells. These cells can act as ‘Trojan horses’ trafficking the virus through the maternal bloodstream and enabling immune evasion [78]. This evasion occurs because undifferentiated myeloid cells establish latent infection characterised by repression of lytic gene expression [79,80]. The infected monocytes may extravasate into the decidua in response to chemokine signals such as CCL2 and CXCL10, where differentiation into macrophages and monocyte-derived DCs enables reactivation of the latent virus and productive replication. Additionally, infected maternal monocytes found in the intervillous space may adhere to the STB, which constitutes the outermost layer of the villi.

Human trophoblasts were historically considered permissive to HCMV because classic owl’s-eye viral inclusions were observed in term placental tissue. However, although trophoblasts have been found to co-stain with CMV immediate early antigens, these characteristic inclusions have not been definitively identified within trophoblasts themselves. Instead, they appear to be a feature of infected stromal and endothelial cells. Notably, even in severely infected placentas, there are studies that have failed to detect HCMV within trophoblast populations [81]. These observations support the view that trophoblasts, particularly STB, are resistant to productive HCMV replication.

**Figure 3:**
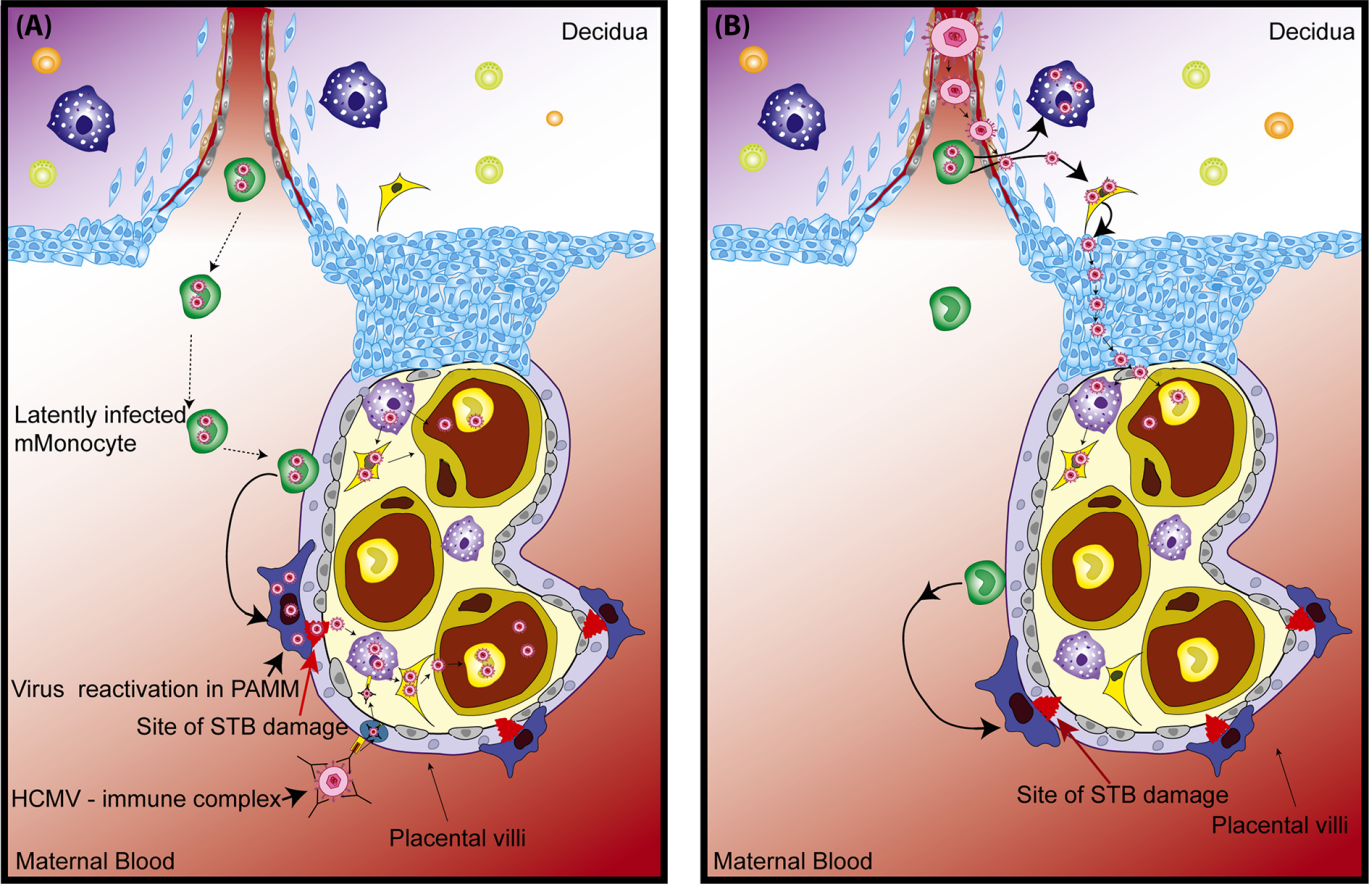
Pathways of HCMV transmission across the maternal–fetal interface (**A**) Hematogenous transmission: Latently HCMV-infected maternal monocytes (green) circulating in the maternal bloodstream may differentiate into PAMM at sites of STB damage (jagged red regions). Upon differentiation into PAMM, these cells can undergo viral reactivation. Sites of STB damage may facilitate viral entry into the placental compartment. HCMV immune complexes may access the placenta through Fc receptors. (**B**) Cell-associated transmission pathway: During HCMV reactivation or primary infection, the virus may directly infect endothelial cells and EVT, followed by decidual cells such as macrophages and fibroblasts. Through cell-to-cell spread, HCMV may progress to the trophoblast cell column and ultimately reach the villous core. Additionally, if a latently infected maternal monocyte enters the decidua and differentiates into a macrophage, this provides an opportunity for viral reactivation. Black arrows indicate the direction of viral spread, and HCMV is represented as red virions. The decidua, STB layer, villous stroma, and placental villi are anatomically distinguished, with maternal blood shown at the base of each panel. HCMV is red/pink.

STBs exhibit several features that ensure their resistance to HCMV infection. While they express epidermal growth receptor (EGFR), which can participate in HCMV entry signalling, they lack essential integrin co-receptors, required for viral attachment and fusion, contributing to their resistance to HCMV infection [82,83]. The dense multinucleated STB layer also lacks intercellular junctions and consists of a dense network of actin filaments preventing viral attachment, entry, and invasion [84]. STB also secretes antimicrobial peptides, extracellular vesicles containing antimicrobial microRNAs [85], and antiviral IFN-λ cytokine [86,87]. These distinctive physical and immunological features make STB remarkably resistant to HCMV infection. However, the integrity of the STB layer is not absolute *in vivo*. Focal disruptions and microtears in the syncytium expose the underlying villous stroma directly to maternal blood [88], potentially enabling HCMV-infected circulating monocytes and PAMM to access the permissive cells of the placental stroma. This is widely accepted to be the most likely route for HCMV transmission to the placental core (Figure 3A).

It has also been proposed that HCMV may reach the placental core through the decidua and the CTB column via cell-to-cell spread [77,89] (Figure 3B). Consistent with this model, confocal microscopy analyses of tissue explant cultures indicate that HCMV can infect maternal decidual macrophages via cell-to-cell transmission [90]. Analyses of *in utero* HCMV-infected decidual sections reveal active viral replication within uterine vasculature, glandular epithelium, and stromal fibroblasts of the decidualised endometrium [77]. α1β1 and αVβ3 act as coreceptors for HCMV entry into fibroblasts, monocytes, and trophoblasts by interacting with HCMV’s glycoprotein B (gB) and the pentameric complex (PC) [91–93]. Due to the invasive behaviour of EVTs and their proximity to infected maternal blood and immune cells, they are anatomically well positioned to encounter maternal viraemia-derived virus. However, evidence from *in vitro* infection assays using organoid models indicates that EVTs highly express antiviral genes and display resistance to productive infection [94]. Through the decidua, the virus can transfer directly to the CTB column through cell-to-cell spread. CTBs express EGFR and integrins α1β1 and αVβ3 that enable virion attachment to distal columns and anchoring villi [92,95]. This route would bypass the STB barrier and allow virus access to the fetal-derived placental compartments indirectly via maternal decidual infection. However, the extent to which such lateral spread occurs *in vivo* remains uncertain.

A study utilising placental explants demonstrated, via immunostaining and PCR, that HCMV IgG immune complexes leverage FcR receptor-mediated transcytosis to cross the syncytium and reach CTBs, where they may be subsequently captured by permissive HBCs within the placental villi [83]. From HBCs, HCMV can potentially spread to stromal fibroblasts and endothelial cells of fetal capillaries in the villus core [77,83]. Infection of villous capillary endothelium would enable viral entry into fetal blood through the capillary network. Once in fetal circulation via the umbilical vein, HCMV disseminates to organs such as the liver, kidneys, and critically, the developing brain and inner ear [81,96,97]. Within the brain, HCMV targets resident neural cells, causing widespread histopathology and inflammation that can result in central nervous system (CNS) sequelae, while infection along the auditory pathway may result in sensorineural hearing loss [96,97].

## Decidual and placental cell responses to HCMV

**Figure 4:**
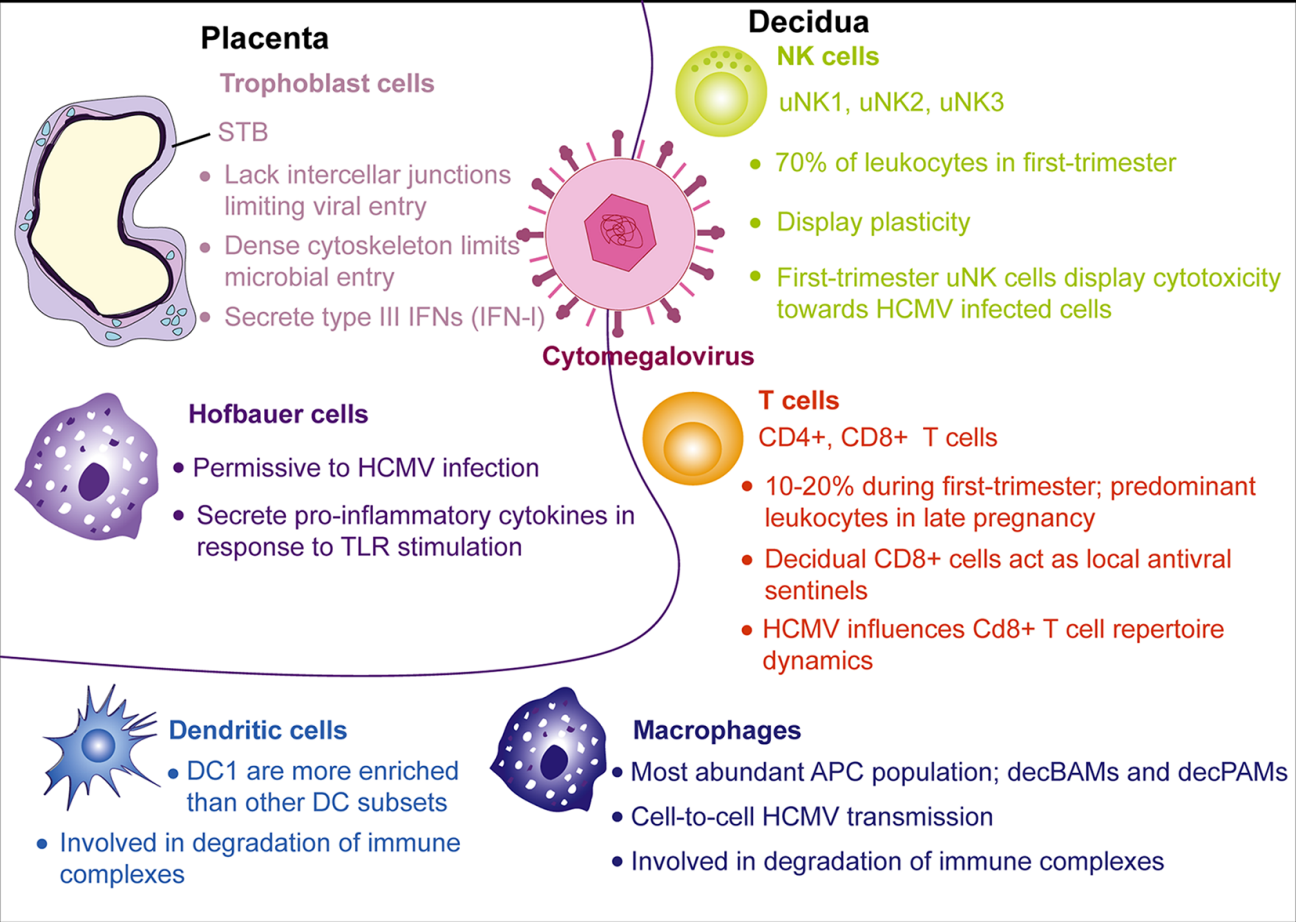
Immune defensive properties of cells at the maternal–fetal interface towards HCMV infection STB restricts viral entry through the continuous multinucleated layer, absence of intercellular junctions, dense cytoskeleton, and constitutive secretion of type III interferons. HBCs are permissive to HCMV infection and initiate proinflammatory responses upon infection. uNK cells exhibit functional plasticity and can mount cytotoxic responses against infected targets. Tissue resident CD8^+^ effector memory T cells rapidly establish an antiviral state following HCMV reactivation. Decidual macrophages (decBAMs and decPAMs) and DCs likely contribute to immune complex clearance through phagocytosis.

Below, we explain the various immune defence mechanisms that cells at the maternal–fetal interface mount against HCMV infection, with a summary provided in Figure 4.

### HCMV and NK cells

NK cells play a critical role in eliminating HCMV-infected cells across tissues (reviewed comprehensively in [98]), prompting longstanding interest in their response to HCMV infection in the decidua, where they exhibit reduced cytotoxicity under steady-state conditions. Correlative evidence suggests that the limited vertical transmission of HCMV during the first trimester coincides with the high abundance of uNK cells, implying a potential protective function [99]. Direct evidence emerged in 2013, when it was demonstrated that uNK cells are involved in controlling viral infections [100]. Using *in vitro* assays with uNK cells isolated from first trimester decidua basalis (8–12 weeks of pregnancy), remarkable plasticity following HCMV exposure was observed, including changes in composition, phenotype, secretome, and cytotoxic effector responses. Specifically, HCMV exposure reduced cytokine-secreting CD56^bright^ uNK cells while increasing CD56^dim^ cells, consistent with the acquisition of the cytotoxic profile. uNK cells exposure to HCMV-infected targets also up-regulated activating receptors (NKp44, NKG2D, and CD94/NKG2C or NKG2E) and down-regulated inhibitory receptors KIR2DL1 and ILT2 [100]. Cytotoxicity was mediated through immunological synapse formation, delivery of perforin- and granzyme-containing lytic granules, and CD107a degranulation, independently of FasL and TRAIL pathways [100]. Notably, term uNK cells exhibit markedly reduced functional activity compared with first trimester cells [101], likely reflecting differences in the abundance of uNK cells subsets (uNK1, uNK2, and uNK3) recently described in early and late gestation [49,50]. The specific antiviral role of these subsets remains to be determined.

Although the specific responses of uNK1, uNK2, and uNK3 subsets have not yet been studied in detail, donor-dependent differences have been observed in relation to KIR2DS1 expression. KIR2DS1^pos^ uNK cells exhibit an enhanced cytotoxic response to HLA-C2^pos^ HCMV-infected maternal decidual stromal cells compared with uNK from KIR2DS1^neg^ donors. Notably, this enhanced response is absent when uNK cells are exposed to HCMV-infected primary EVTs [102]. HCMV-induced down-regulation of HLA-C on EVT cells may facilitate immune evasion [102,103]. NK cells may also harness the antiviral mechanisms of neighbouring immune cells to clear HCMV-infected cells. For instance, uNK cells exposed to HCMV-infected fibroblasts up-regulated HLA-DR expression, which could lead to T cell activation and thereby impaired viral spread [100].

Research investigating the role of uNK cells in HCMV interactions is still emerging, and several key questions remain unanswered: (i) Do any uNK cell subsets utilise Fc-mediated antibody dependent cellular cytotoxicity to recognise and kill HCMV-infected cells? (ii) Can uNK cells expand in response to HCMV infection and generate ‘adaptive or memory-like’ properties as observed in peripheral NK cells following HCMV infection or reactivation in healthy individuals and transplant patients [104]? (iii) Does prior HCMV exposure influence the response of uNK cells to a secondary HCMV challenge or reactivation?

### HCMV and T cells

HCMV serostatus can profoundly influence the repertoire of circulating CD8^+^ T cells and its dynamics during pregnancy. In HCMV seropositive healthy pregnant women, the CD8^+^ T cell phenotype shifts over the course of pregnancy, with a 50% reduction in the naïve CCR7^+^ CD45RA^+^ CD8^+^ T cells and a corresponding increase in effector memory CCR7^−^ CD45RA^+^ CD8^+^ T cells [105]. Using an *ex vivo* human decidual organ culture model of HCMV infection, decidual tissues from women with pre-existing CMV immunity display an intrinsic resistance to the virus, characterised by rapid activation of tissue-resident memory CD8^+^ and CD4^+^ T cells upon reinfection. Moreover, HCMV-specific-tissue resident CD8^+^ effector memory T cells in the decidua may function as local sentinels, swiftly establishing an antiviral state at the maternal–fetal interface following HCMV reactivation [106].

A moderate increase in invariant NKT (iNKT) cells was detected in HCMV-infected decidua [107]. iNKT cells represent a specialised T cell subset that eliminates virus-infected cells in a CD1d-dependent manner [108]. Further investigation is required to understand the role of iNKT cells in congenital HCMV infection.

### HCMV and decidual macrophages

Decidual macrophages are the major mononuclear phagocyte population in the decidua, accounting for 20% of the total immune cells [109]. HCMV can infect the maternal decidual macrophages via cell-to-cell transmission [90]. Both macrophages and DCs in basal decidua express FcγRIIA and FcRn that cooperate during binding and degradation of immune complexes for antigen presentation and activation of T cells [107]. However, their interaction with HCMV has not been studied in depth.

### HCMV and Hofbauer cells

Given the challenges associated with isolating purified HBC for *in vitro* assays, the most robust evidence regarding these cells in the context of HCMV infection derives from *in situ* microscopy. Studies have shown that HBCs exhibit hyperplasia *in situ* during congenital HCMV infection [110,111]. Analysis of tissue sections from *in utero* infections has revealed HCMV proteins localised within cytoplasmic vesicles, along with the production of proinflammatory cytokines and type I IFNs by HBCs [77,110,112–114]. A vaccine study demonstrated that neutralising antibodies targeting the pentamer complex can reduce HCMV infection in rhesus macaque placental macrophages [115]. However, it remains unclear whether HCMV actively replicates in HBCs or whether these cells are merely permissive to infection. Furthermore, the extent to which HBCs are routinely infected during placental infection is uncertain, as findings are inconsistent. Some investigations reported an absence of HBC infection based on immunohistochemical analysis of placental tissue from HCMV-infected women [81], whereas others detected viral DNA within these cells [111,112].

### Trophoblast-specific defences

The placenta serves as a critical immunological barrier, protecting the developing fetus from pathogenic invasion. Its specialised microarchitecture underpins several antiviral defences. STBs form a continuous, multinucleated layer devoid of intercellular junctions and reinforced by a dense cytoskeleton, thereby limiting microbial entry. STB constitutively secrete type III IFNs (IFN-λ) and vesicle encapsulated primate-specific placental microRNAs (miRNAs) [chromosome 19 miRNA cluster (C19MC)], which restrict viral infections through autocrine and paracrine mechanisms [85,116]. Additionally, high basal autophagy rates in STB provide a broad intracellular antimicrobial mechanism against diverse pathogens [85]. Trophoblast cells also mount innate immune responses by recognising pathogens via Toll-like receptors, which activate the induction of antimicrobial signalling pathways [117]. Due to the difficulty of studying STB *in vitro*, its specific interactions with HCMV remain poorly described. More general trophoblast-specific defence mechanisms have been reviewed in detail elsewhere [118–120].

### Additional aspects for development

Further studies should explore HCMV interactions with B cells (1%–2% of decidual lymphocytes) [121] and minor immune subsets, including innate lymphoid cells [122], MAIT cells [123] and DCs [124], to elucidate their roles in vertical transmission and placental immunopathology. Additionally, HCMV can infect placental pericytes in a lytic manner, disrupting vascular integrity and promoting inflammation, which may increase fetal exposure during maternal viraemia [125]. Another intriguing question is how human endogenous retroviruses, many of whose genes are expressed in the placenta and have contributed to its evolutionary development, interact with decidual immune cells and thereby shape immune responses to HCMV during pregnancy [126].

## HCMV evasion of immune defences

HCMV, a ‘master of immune evasion’, has evolved mechanisms to evade both innate defences and adaptive immune responses, including cell-mediated and humoral components, at the maternal–fetal interface.

### Innate immune evasion and immunopathology

HCMV dampens the innate immune response during the early stages of lytic infection by targeting intrinsic immunity, as well as NF-kB and IFN signalling. The immediate early protein, IE1, produced immediately upon infection, binds and sequesters STAT2, preventing up-regulation of interferon-stimulated genes (ISGs) [127]. IE86 acts as an NF-kB antagonist and mediates proteosome-dependent STING degradation, thereby inhibiting the cGAS-STING pathway [128]. Because IFN signalling pathway is a potent barrier to HCMV pathogenesis, the virus has evolved multiple strategies to modulate this response. IE86 obstructs NF-kB interaction with the IFN-β promoter [129,130], while US9 interferes with type I IFN signalling and IFN-β production [131]. In addition, HCMV proteins can target specific ISGs, such as UL26, which interacts with ISG15 (a ubiquitin-like protein) and blocks its activation [132]. The APOBEC3 family member APOBEC3A (A3A), an interferon inducible antiviral factor, is up-regulated in the maternal decidua upon HCMV infection or IFN-β stimulation and restricts viral replication [133]. To counteract this, HCMV exhibits mutational robustness by depleting APOBEC3G (AG3) ‘hot spot’ motifs from essential ORFs, thereby enabling escape from IFNβ-driven cytosine deamination and replication inhibition [134]. It is also plausible that HCMV has evolved mechanisms to circumvent A3A restriction, but that remains to be determined.

HCMV has evolved sophisticated mechanisms to manipulate the host chemokine and cytokine milieu, promoting increased lymphocyte trafficking to the maternal–fetal interface and thereby disrupting the immune privileged environment of the decidua. *In vitro* studies demonstrate that HCMV infection elicits a rapid and robust innate immune response in decidual tissue, characterised by pronounced induction of IFN-γ and CXCL10 (IP-10) induction, which markedly alters the local cytokine and chemokine landscape [135]. Furthermore, *in vitro* infection of CTBs with HCMV results in dysregulated cell-matrix and cell–cell adhesion activity, including reduced matrix metalloproteinase MMP-9 production mediated by HCMV-IL-10, a viral cytokine with immunosuppressive activities that impairs CTB invasiveness [95]. Another study demonstrated that pregnant women with HCMV fetal infection had elevated serum CXCL10 and elevated amniotic fluid levels of TNF-α, IL-1β, IL-10, IL-12, IL-15, IL-17, CCL2, CCL4, and CXCL10 [136]. Additionally, HCMV infection enhances collagen deposition through integrin-αvβ6-mediated activation of TGF-β in endothelial cells, potentially compromising uteroplacental blood flow [137]. Collectively, these alterations underscore the multifaceted impact of HCMV on placental immune regulation and vascular integrity, highlighting critical pathways that may drive vertical transmission and adverse pregnancy outcomes.

### Adaptive immune evasion

HCMV interferes with all stages of the MHC class I antigen presentation pathway to avoid CD8^+^ T cell recognition by encoding the US6 family members, namely US2, US3, US6, and US11 (reviewed comprehensively in [138]). However, down-regulation of MHC class I or other ‘self’ markers could make the virus-infected cells susceptible to NK cell-mediated cytotoxicity via the ‘missing self’ recognition [103]. After HCMV infection, EVT continued to express the MHC class I molecules HLA-C, -E, and -G. This may represent a strategy to protect HCMV-infected cells from being recognised and attacked by uNK cells, thereby preventing a large-scale maternal immune response against the placenta during infection [139].

EVTs modulate uNK cell activity through expression of HLA-E and HLA-G, engaging CD94/NKG2A/C and LILRB1 receptors, respectively [140]. HCMV has evolved immune evasion strategies that mimic or replace host MHC class I molecules to engage inhibitory NK cell receptors and suppress NK cell-mediated cytotoxicity. The viral glycoprotein, UL40, stabilises HLA-E surface expression by loading UL40 peptide, thereby protecting infected cells from lysis via engagement of CD94/NKG2A^+^ NK cells [141]. Notably, UL40 peptide polymorphisms can skew signalling towards inhibitory NKG2A or activating NKG2C, influencing outcomes in congenital infection [142,143]. Additionally, the viral MHC class I homologue, UL18, exhibits high affinity to inhibitory receptor LILRB1, outcompeting the host MHC and further dampening NK activation, a mechanism particularly relevant in decidua where LILRB1 is coexpressed on uNK cells alongside trophoblast HLA-G [144–146]. Together, these adaptations illustrate how HCMV exploits NK cell receptor pathways to maintain persistence at the maternal–fetal interface, thereby facilitating vertical transmission.

The specialised maternal–fetal IgG transport pathway provides a potential route for HCMV transmission. During early infection, when low-avidity IgG antibodies predominate, HCMV can exploit antibody-dependent enhancement (ADE) to bypass humoral immunity and traverse the STB [83,147]. IgG virion complexes may undergo transcytosis across STB without complete neutralisation reaching the basal membrane where they encounter underlying CTBs expressing viral entry receptors. Additionally, HBCs, which express Fc receptors, can internalise these complexes, establishing focal infection that facilitates cell-to-cell spread to stromal fibroblasts and villous capillaries within the fetal compartment, as observed in infected term placentas [83,148]. These findings highlight how HCMV co-opts physiological IgG transport mechanisms to breach placental barriers, underscoring a critical pathway for vertical transmission.

## HCMV diagnosis and clinical interventions

Screening for maternal CMV infection in pregnancy varies by global context. While some European settings implement universal screening for CMV in early pregnancy, the U.K. adopts a more targeted approach, investigating evidence of HCMV seroconversion only in women with a high risk of infection such as pregnant women who work in daycare centres [149–151]. In parallel, a subset of countries has incorporated high-dose valaciclovir treatment as a recommended intervention following primary maternal infection [152]. In regions where routine screening is not implemented, this largely reflects the ongoing challenge that effective interventions to prevent *in utero* transmission or mitigate associated sequelae remain limited. Furthermore, using available diagnostic methods, it is challenging to reliably identify women who have seroconverted to HMCV during pregnancy and are thus a higher risk group for fetal transmission. Compounding these challenges, there is no licensed treatment to protect seronegative mothers from primary HCMV infection during pregnancy.

Most maternal HCMV infections remain asymptomatic. Diagnosis of primary maternal HCMV infection relies on serological testing for HCMV-specific IgG and IgM antibodies [152]. Women with positive IgM antibodies in combination with a low avidity IgG response are usually assumed to have contracted a primary HCMV infection during the pregnancy [153]. Results from routine blood tests (taken at 11–14 weeks gestation) can be compared with those from later in the pregnancy to give the best indication of when seroconversion may have occurred [152,154]. Confirmation of CMV seropositivity requires PCR testing of maternal blood samples [155,156]. Detailed ultrasound for detection of associated anomalies is undertaken when HCMV infection is suspected [157]. Recent evidence suggests that a negative viral PCR in placentas obtained through chorionic villus sampling after primary infection is reassuring with respect to fetal outcome [158].

Although the development of HCMV vaccines has been designated a top priority by the National Vaccine Advisory Committee [159], no licensed vaccine exists to prevent HCMV infection. Key challenges for vaccine development include the following: (a) achieving efficacy beyond the ∼50% protection against maternal infection observed in previous trials, and (b) generating evidence that demonstrates protection against fetal infection and its sequelae, even if maternal immunity is incomplete. Current candidate vaccines under evaluation include vaccines targeting envelope glycoproteins such as gB and the pentameric complex (PC), as well as formulations incorporating gB with nonenveloped virion proteins and non-structural proteins to elicit both neutralising antibodies and robust CD4/CD8^+^ T cell responses. Previous trials using recombinant gB vaccine in HCMV seronegative women achieved only 45%–50% efficacy for preventing seroconversion, with immunity waning over time [160,161]. The recent phase III trial of the Moderna mRNA 1647 vaccine that codes for both gB and the PC [162] failed due to poor efficacy. Multiple additional vaccine platforms remain in preclinical and clinical development and are reviewed elsewhere [163].

Currently, the only pharmacological intervention for maternal HCMV infection is high dose oral valaciclovir. As a prodrug of aciclovir, valaciclovir exhibits activity against HCMV DNA polymerase when administered at 8 g/day, significantly reducing the risk of congenital infection following primary maternal infection [164,165]. This dose is considerably higher than that used for other indicators and is guided by pharmacokinetic data demonstrating that such elevated dosing is required to achieve sufficient transplacental transfer of the active compound to the fetal compartment. While uncertainties regarding valaciclovir’s efficacy and safety have resulted in limited widespread clinical adoption, the latest guidelines from the Royal College of Obstetricians and Gynaecologists recommend that oral valaciclovir treatment may be offered to reduce the risk of transplacental transmission when administered soon after maternal primary CMV infection in the first trimester [166].

For neonates diagnosed with congenital HCMV involving the CNS, first-line therapy consists of nucleoside analogue ganciclovir and its prodrug valganciclovir, typically administered for 6 months [154]. This treatment was shown to reduce hearing loss in five out of six babies and improve long-term brain development outcomes in some [166]. Further investigation into antiviral safety, teratogenicity, and novel therapeutic candidates is essential to improve maternal and neonatal outcomes in congenital HCMV infection. Other compounds that are being tested are discussed comprehensively elsewhere [167].

Despite advances in diagnostic techniques and promising vaccine and therapeutic candidates, the absence of routine screening, reliable interventions for nonprimary infections, and universally effective preventive strategies underscores the urgent need for continued research to reduce the burden of congenital HCMV.

## Model systems and viral strain selection for studying HCMV at the maternal–fetal interface

### HCMV strains

HCMV strains exhibit extensive genetic diversity that influences viral transmissibility, cellular tropism, and clinical outcomes. Accurate investigation of HCMV pathogenesis requires the use of clinical strains, such as Merlin and TB40/E, which retain an intact UL128-UL151 (ULb') region encoding immune evasion genes. These clinical strains also preserve the capacity of cell-to-cell spread, the predominant mode of HCMV dissemination *in vivo* [168,169]. In contrast, high-passaged laboratory strains AD169 and Towne exhibit significant genetic deletions and rearrangements, resulting in compromised pathogenicity, altered cellular tropism, and a shift toward cell-free spread [170].

### Animal models

Animal models have provided valuable insights into CMV disease and congenital infection. However, as CMV is species-specific, findings must be interpreted with caution. Animal models of congenital infection differ from human pregnancy in key aspects of placental architecture, notably the transition from yolk sac to haemochorial placentation and the number of trophoblast invasion layers, which may alter the route, efficiency, and outcome of vertical transmission [171–173]. Species-specific differences in innate and adaptive immune responses at the maternal–fetal interface including the composition of uNK cells further limit direct extrapolation of immune correlates of protection to human pregnancy [36]. Murine models have been important for understanding CMV pathogenesis, particularly mechanisms of brain injury and sensorineural hearing loss [174,175]. Unlike HCMV, murine CMV does not cross the placenta [176], and strain-specific differences in susceptibility driven by NK cell effector responses [177,178] limit their utility for studying CMV–uNK cell interactions. The guinea pig CMV (GPCMV) model offers advantages, as GPCMV can cross the placenta and often induces histopathological changes resembling those seen in human placentas exposed to HCMV *in utero* [179–181]. Nonhuman primate (NHP) models, such as rhesus macaques, have also been employed; direct inoculation of RhCMV into fetuses during the first and second trimesters produces brain lesions and neurologic sequelae similar to those seen in infants with congenital HCMV, underscoring the clinical relevance of this model [182,183]. Nevertheless, both guinea pig and NHP models are technically challenging to establish and lack genetic tractability, restricting mechanistic studies.

### Explant models

**Human placental explants** derived from early to late gestation and cultured on specialised substrates, recapitulate key elements of placental differentiation and invasion. These explants preserve the main architecture of chorionic tissue and enable multicellular cross-talk, as they comprise diverse cell types including mesenchymal cells, endothelial cells, and immune cells [184]. A pioneering study conducted in 1987 demonstrated that first-trimester human placental explants are permissive to HCMV infection *in vitro* and support latent and/or abortive HCMV infection [185]. HCMV can undergo multiple cycles of replication within explants, progressing from infected trophoblast cells and spreading cell-to-cell into stromal fibroblasts, before ultimately reaching fetal endothelial cells [186]. Placental explants have been widely used to investigate HCMV pathogenesis, viral transmission and replication in CTBs and STBs [76], gestational differences in permissiveness [187], modulation of production of cytokines and proinflammatory mediators [188,189], effects on placental small extracellular vesicles (sEVs) [190], and the impact of neutralising antibodies and hyperimmune on viral replication [191,192]. Although explants partially mimic the *in vivo* placental environment, their use has declined due to significant limitations: short viability (with syncytium detachment within 24 h), high inter-individual variability, lack of scalability, and poor genetic tractability [193].

**Decidual tissue slices** are another valuable *ex vivo* model that preserves tissue architecture and immune cell populations. This system has been used to study multiple aspects of HCMV pathogenesis, including the impact of primary versus nonprimary infection on local immune responses [106] and the dynamics of viral transmission at the maternal–fetal interface [90]. However, it faces similar limitations to placental villi explant models.

### *In vitro* trophoblast cell models

Understanding HCMV cell tropism, genetic diversity, and replication dynamics provided the basis for developing the first *in vitro* placental models in 1981 [194]. Since then, a range of experimental systems, including villous explants and monolayer and three-dimensional (3D) cell models, have been employed to study HCMV pathogenesis.

**Cell lines** such as BeWo, JEG-3, and JAR cells, derived from choriocarcinomas, are widely used to investigate various aspects of trophoblast biology. They have served as models for studying trophoblast fusion [195,196], drug transport [197], and placenta-derived pregnancy disorders including pre-eclampsia and recurrent miscarriage [198]. Although choriocarcinoma cell lines have been used to model HCMV infection in the placenta, several limitations exist. These cell lines originate from tumours rather than healthy trophoblast cells; therefore they do not fully recapitulate normal trophoblast biology. For example, JEG-3 cells support productive HCMV replication, whereas JAR cells fail to do so [199]. Additional models include immortalised EVT cell lines such as HTR-8/SVneo and SGHPL-4 [200–203], as well as Swan 71, a CTBic-derived cell line [204]. While in the past, these systems have provided valuable insights into HCMV placental interactions, their antiviral properties can differ from those of primary trophoblasts, limiting their utility as infection models [86].

**Cultured primary human trophoblast** (PHT) are *in vitro* preparations of purified trophoblast cells freshly isolated from human placental tissue [205]. PHT cultures enable the study of distinct trophoblast subtypes, including cytotrophoblasts and STB, and have facilitated the characterisation of key regulatory genes in the trophoblast fusion (reviewed in [206]). Despite their intrinsic antiviral defences, certain trophoblast subtypes can be infected by and support viral replication [207]. CTBs appear more susceptible to HCMV infection than STBs, with the virus shown to inhibit CTB syncytialisation [208]. However, PHTs present notable challenges: they are prone to contamination by stromal and blood cells, lack efficient methods for genetic manipulation, and exhibit a limited life span in culture (3–5 days), restricting their experimental utility.

**2D stem cell models** [209] **and 3D trophoblast organoids** are two recent *in vitro* systems derived from first trimester and term placental tissues [210–212]. These approaches enable detailed investigation of trophoblast biology and differentiation under controlled conditions. Both models preserve authentic trophoblast lineage identity, support long-term expansion and offer genetic tractability, making them highly suitable for infection assays. Compared with short-lived explants or transformed cell lines, these systems provide greater reproducibility and mechanistic insight into placental development and pathogen interactions [213].

Human TSCs represent an emerging *in vitro* model for studying HCMV infection mechanisms in trophoblast cells. These cells can be maintained long-term or induced to differentiate into either STB or EVTs [209]. Historically, chorion-derived trophoblast progenitor cells, likely the closest counterpart to TSCs, were used to investigate HCMV infection and demonstrated productive infection, although trophoblast cells derived from the smooth chorion may be committed to a different cell fate than villous trophoblast cells [214–217]. HCMV was found to non-productively infect TSCs as well as TSC-derived EVTs and STB [207], a finding that contrasts with earlier reports of productive infection in CTBs [208].

The recent development of trophoblast organoids has significantly expanded the experimental models available for studying HCMV interactions at the human maternal–fetal interface [211]. Trophoblast organoids are 3D cell cultures that closely mimic the microarchitecture and cellular dynamics of the placenta *in vitro* [210,211]. Their structural organisation resembles the villous placenta with a layer of CTBs that differentiate into STB [212]. Trophoblast organoids can be refractory to HCMV infection, partly due to constitutive secretion of type III interferon, whereas decidual organoids are permissive to both HCMV laboratory strain AD169r and clinical strain TB40E [218]. Although organoid models represent a major advance for studying human trophoblast biology, they do have limitations: they recapitulate only the trophoblast component and lack vascular and HBC cells, which may play critical roles in placental development and function. Future efforts should include creating more complex, physiologically relevant models that integrate multiple placental cell types to better capture *in vivo* conditions.

Analyses of placental sections from *in utero* HCMV infection clearly demonstrate the presence of viral proteins within CTB [83]. However, whether these cells support productive infection remains uncertain. *In vitro* studies have not resolved this question, as outcomes appear to vary depending on the culture system employed.

The integration of organ-on-a-chip technology with organoid systems has recently led to the development of organoid-on-chip platforms, which offer enhanced physiological relevance and tractable microenvironments [219,220]. The placenta-on-a-chip model enables the reconstruction of the multilayered placental barrier and simulation of hemodynamic conditions [221]. Although this approach has been applied to other transplacental pathogens [222], HCMV infection has not yet been investigated using this system.

**Figure 5:**
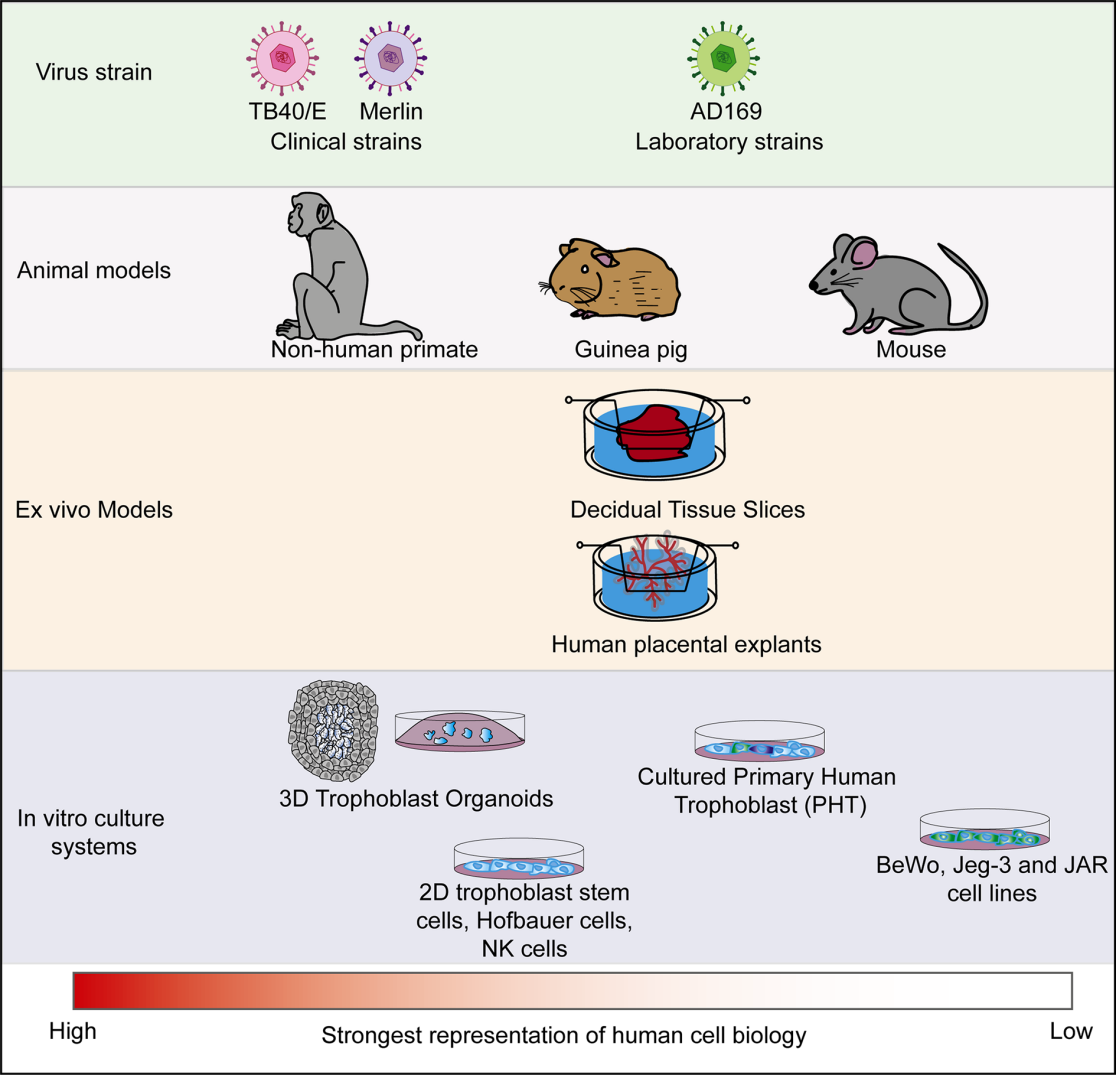
Experimental models for studying HCMV infection, organised by their physiological relevance to human biology The top row shows representative HCMV strains, including clinical strains (e.g., TB40/E and Merlin) and laboratory strains (e.g., AD169). Below, three categories of experimental models are depicted. Animal models include nonhuman primates, guinea pigs, and mice. *Ex vivo* models comprise decidual tissue slices and human placental explants, which maintain native tissue architecture. *In vitro* culture systems include 3D trophoblast organoids and 2D trophoblast stem cells, HBCs, and NK cells cultured with PHT, as well as established cell lines (BeWo, Jeg-3, and JAR). The colour gradient from red (high) to white (low) at the bottom indicates the strength of representation of primary human placental biology and HCMV infection.

Figure 5 summarises experimental models used to study HCMV infection, ranging from animal models and *ex vivo* tissue systems to *in vitro* culture models organised along a gradient reflecting how accurately each recapitulates primary human placental biology and HCMV infection.

### *In vitro* immune cell models

#### Hofbauer cells

Multiple protocols have been employed to isolate HBCs from first- and term-placental digests, including enzymatic digestion, density gradient centrifugation (Pancoll and Ficoll), magnetic bead separation (MACS), and fluorescence-activated cell sorting (FACS). However, achieving high-yield, high-purity preparations free from maternal and fetal blood contaminants, as well as decidual cells, remains challenging. Such contamination can significantly affect the interpretation of HBC responses and their permissiveness to HCMV. Our laboratory previously demonstrated that incorporating antibodies to common HLA allotypes enables discrimination between maternal and fetal cells, ensuring a pure HBC population [58,223]. Additionally, HBC, like all macrophages, is sensitive to changes in its local microenvironment [45], and this must be considered when analysing experimental outcomes.

#### Uterine NK cells

uNK cells have traditionally been studied as a whole population using *in vitro* HCMV infection assays as described above. While informative, a major challenge is ensuring complete removal of blood contaminants, as many studies do not clearly describe purification methods or provide data on cell purity [100]. Advances in FACS now enable the isolation of uNK cell subsets (uNK1, uNK2, and uNK3) for *in vitro* studies [49,52,224]; however, to our knowledge, this approach has not yet been applied in the context of HCMV since these subsets were first defined. More recently, iPSC-derived NK cells have been integrated with organoid platforms, an emerging strategy that may provide a physiologically more relevant system for advancing our understanding of uNK cell biology and its role in HCMV infection [225].

## Confounding host and viral factors

The central challenge in understanding congenital HCMV infection is that not all infections result in clinical disease. Around 10%–15% of infants with congenital CMV exhibit symptoms at birth, whereas the remaining 85%–90% are asymptomatic [226]. The determinants of whether infection manifests as asymptomatic or pathogenic are multifactorial and complex. Maternal immune status is a key factor, as is the infant’s genetic background, including HLA type and polymorphisms in innate immune genes [227]. The timing and nature of maternal infection, such as primary versus non-primary infection and first versus third trimester exposure, also critically influence outcomes.

Viral factors add another layer of complexity. Both HCMV strain type and strain diversity must be considered. Within the maternal host, numerous HCMV strains circulate, and mixed infections, where multiple strains coexist, potentially lead to the transmission of one or, less commonly, several strains to the fetus, though in most instances a single strain is transmitted, suggesting a transmission bottleneck [228].

Evidence indicates that all gB and UL144 genotypes are capable of maternal-to-fetal transmission and can influence viral burden (reviewed in [229]). Placental factors, including differential expression of HCMV receptors between STB and CTBs [88] as well as the local immune microenvironment at the fetal–maternal interface, further modulate virus access to the fetus and the severity of infection. Importantly, not all determinants are known; some factors may be stochastic or subtle in nature.

Continued research is essential, with a focus on developing improved experimental models and deeper investigation into HCMV pathogenesis to guide the development of targeted interventions, including vaccines, aimed at mitigating adverse outcomes in pregnant women and neonates. More broadly, elucidating the immunological dynamics at the maternal–fetal interface through clinically relevant *in vitro* models will be critical to enable safe and responsible inclusion of pregnant women in clinical trials of vaccines and therapeutics against HCMV and other transplacental pathogens. Additionally, enhancing pregnancy outcomes will require more sensitive diagnostic tools capable of detecting lytic HCMV infection before fetal development is compromised.

## Open Access

Open access for this article was enabled by the participation of University of Cambridge in an all-inclusive Read & Publish agreement with Portland Press and the Biochemical Society under an agreement with JISC.

## Abbreviations

3D: three-dimensional
CTB: cytotrophoblast
DCs: dendritic cells
decBAMs: decidua basalis-associated macrophages
decPAMs: decidua parietalis-associated macrophages
EVT: extravillous trophoblast
gB: glycoprotein B
GPCMV: guinea pig CMV
HCMV: human cytomegalovirus
HBCs: Hofbauer cells
ISGs: interferon-stimulated genes
iNKT: invariant NKT
miRNAs: microRNAs
NHP: nonhuman primate
ORFs: open reading frames
PAMM: placental-associated maternal macrophages
PCP: pentameric complex
PHT: primary human trophoblast
STB: syncytiotrophoblast
uNK: uterine natural killer

## References

[1] Ribbert H. (1904) Uber protozoenartige Zellen in der Niere eines syphilitischen Neugeborenen und in der Parotis von Kindern. Zbl. Allg. Pathol. 15, 945–948

[2] Goodpasture E.W. and Talbot F.B. (1921) Concerning the nature of protozoan-like cells in certain lesions of infancy. Am. J. Dis. Child. 21, 415–425 10.1001/archpedi.1921.01910350002001

[3] Smith M.G. (1956) Propagation in tissue cultures of a cytopathogenic virus from human salivary gland virus (SGV) disease. Proc. Soc. Exp. Biol. Med. 92, 424–430 10.3181/00379727-92-22498 13350368

[4] Rowe W.P., Hartley J.W., Waterman S., Turner H.C. and Huebner R.J. (1956) Cytopathogenic agent resembling human salivary gland virus recovered from tissue cultures of human adenoids. Proc. Soc. Exp. Biol. Med. 92, 418–424 10.3181/00379727-92-22497 13350367

[5] Craig J.M., Macauley J.C., Weller T.H. and Wirth P. (1957) Isolation of intranuclear inclusion producing agents from infants with illnesses resembling cytomegalic inclusion disease. Proc. Soc. Exp. Biol. Med. 94, 4–12 10.3181/00379727-94-22841 13400856

[6] Weller T.H. (1970) Review. Cytomegaloviruses: the difficult years. J. Infect. Dis. 122, 532–539 10.1093/infdis/122.6.532 4321292

[7] Weller T.H., Hanshaw J.B. and Scott D.E. (1960) Serologic differentiation of viruses responsible for cytomegalic inclusion disease. Virology 12, 130–132 10.1016/0042-6822(60)90156-2 13843861

[8] Elek S.D. and Stern H. (1974) Development of a vaccine against mental retardation caused by cytomegalovirus infection *in utero*. Lancet 1, 1–5 10.1016/S0140-6736(74)92997-3 4128996

[9] Plotkin S.A., Furukawa T., Zygraich N. and Huygelen C. (1975) Candidate cytomegalovirus strain for human vaccination. Infect. Immun. 12, 521–527 10.1128/iai.12.3.521-527.1975 170203 PMC415318

[10] Gleaves C.A., Smith T.F., Shuster E.A. and Pearson G.R. (1984) Rapid detection of cytomegalovirus in MRC-5 cells inoculated with urine specimens by using low-speed centrifugation and monoclonal antibody to an early antigen. J. Clin. Microbiol. 19, 917–919 10.1128/jcm.19.6.917-919.1984 6088574 PMC271213

[11] Razonable R.R. (2023) Oral antiviral drugs for treatment of cytomegalovirus in transplant recipients. Clin. Microbiol. Infect. 29, 1144–1149 10.1016/j.cmi.2023.03.020 36963566

[12] Hocker J.R., Cook L.N., Adams G. and Rabalais G.P. (1990) Ganciclovir therapy of congenital cytomegalovirus pneumonia. Pediatr. Infect. Dis. J. 9, 743–745 10.1097/00006454-199010000-00013 2172904

[13] Chee M.S., Bankier A.T., Beck S., Bohni R., Brown C.M., Cerny R. et al. (1990) Analysis of the protein-coding content of the sequence of human cytomegalovirus strain AD169. Curr. Top. Microbiol. Immunol. 154, 125–169 10.1007/978-3-642-74980-3_6 2161319

[14] Dolan A., Cunningham C., Hector R.D., Hassan-Walker A.F., Lee L., Addison C. et al. (2004) Genetic content of wild-type human cytomegalovirus. J. Gen. Virol. 85, 1301–1312 10.1099/vir.0.79888-0 15105547

[15] Puliyanda D.P., Silverman N.S., Lehman D., Vo A., Bunnapradist S., Radha R.K. et al. (2005) Successful use of oral ganciclovir for the treatment of intrauterine cytomegalovirus infection in a renal allograft recipient. Transpl. Infect. Dis. 7, 71–74 10.1111/j.1399-3062.2005.00089.x 16150094

[16] Wang D. and Shenk T. (2005) Human cytomegalovirus virion protein complex required for epithelial and endothelial cell tropism. Proc. Natl. Acad. Sci. U.S.A. 102, 18153–18158 10.1073/pnas.0509201102 16319222 PMC1312424

[17] Stern-Ginossar N., Weisburd B., Michalski A., Le V.T., Hein M.Y., Huang S.X. et al. (2012) Decoding human cytomegalovirus. Science 338, 1088–1093 10.1126/science.1227919 23180859 PMC3817102

[18] Zhang L., Yu J. and Liu Z. (2020) MicroRNAs expressed by human cytomegalovirus. Virol J. 17, 34 10.1186/s12985-020-1296-4 32164742 PMC7069213

[19] Pignatelli S., Dal Monte P., Rossini G. and Landini M.P. (2004) Genetic polymorphisms among human cytomegalovirus (HCMV) wild-type strains. Rev. Med. Virol. 14, 383–410 10.1002/rmv.438 15386592

[20] Mocarski E., Shenk T. and Pass R. (2007) Cytomegalovirus. In Fields Virology, (Knipe D.M. and Howley P.M., eds), pp. 1960–2014, Lippincott Williams & Wilkins, Philadelphia

[21] Nassetta L., Kimberlin D. and Whitley R. (2009) Treatment of congenital cytomegalovirus infection: implications for future therapeutic strategies. J. Antimicrob. Chemother. 63, 862–867 10.1093/jac/dkp083 19287011 PMC2667137

[22] Boppana S.B., Rivera L.B., Fowler K.B., Mach M. and Britt W.J. (2001) Intrauterine transmission of cytomegalovirus to infants of women with preconceptional immunity. N. Engl. J. Med. 344, 1366–1371 10.1056/NEJM200105033441804 11333993

[23] Bodeus M., Hubinont C. and Goubau P. (1999) Increased risk of cytomegalovirus transmission* in utero* during late gestation. Obstet. Gynecol. 93, 658–660 10912962

[24] Enders G., Daiminger A., Bader U., Exler S. and Enders M. (2011) Intrauterine transmission and clinical outcome of 248 pregnancies with primary cytomegalovirus infection in relation to gestational age. J. Clin. Virol. 52, 244–246 10.1016/j.jcv.2011.07.005 21820954

[25] Abu-Raya B., Michalski C., Sadarangani M. and Lavoie P.M. (2020) Maternal immunological adaptation during normal pregnancy. Front. Immunol. 11, 575197 10.3389/fimmu.2020.575197 33133091 PMC7579415

[26] Kenneson A. and Cannon M.J. (2007) Review and meta-analysis of the epidemiology of congenital cytomegalovirus (CMV) infection. Rev. Med. Virol. 17, 253–276 10.1002/rmv.535 17579921

[27] Swanson E.C. and Schleiss M.R. (2013) Congenital cytomegalovirus infection: new prospects for prevention and therapy. Pediatr. Clin. North Am. 60, 335–349 10.1016/j.pcl.2012.12.008 23481104 PMC3807860

[28] Zuhair M., Smit G.S.A., Wallis G., Jabbar F., Smith C., Devleesschauwer B. et al. (2019) Estimation of the worldwide seroprevalence of cytomegalovirus: a systematic review and meta-analysis. Rev. Med. Virol. 29, e2034 10.1002/rmv.2034 30706584

[29] Lanzieri T.M., Dollard S.C., Bialek S.R. and Grosse S.D. (2014) Systematic review of the birth prevalence of congenital cytomegalovirus infection in developing countries. Int. J. Infect. Dis. 22, 44–48 10.1016/j.ijid.2013.12.010 24631522 PMC4829484

[30] Tabata T., Petitt M., Fang-Hoover J., Zydek M. and Pereira L. (2016) Persistent cytomegalovirus infection in amniotic membranes of the human placenta. Am. J. Pathol. 186, 2970–2986 10.1016/j.ajpath.2016.07.016 27638253 PMC5222962

[31] Bialas K.M. and Permar S.R. (2016) The March towards a vaccine for congenital CMV: rationale and models. PLoS Pathog. 12, e1005355 10.1371/journal.ppat.1005355 26866914 PMC4750955

[32] Ssentongo P., Hehnly C., Birungi P., Roach M.A., Spady J., Fronterre C. et al. (2021) Congenital cytomegalovirus infection burden and epidemiologic risk factors in countries with universal screening: a systematic review and meta-analysis. JAMA Netw. Open 4, e2120736 10.1001/jamanetworkopen.2021.20736 34424308 PMC8383138

[33] Mussi-Pinhata M.M., Yamamoto A.Y., Brito R.M.M., Isaac M.D., Oliveira P.F.D.E., Boppana S. et al. (2009) Birth prevalence and natural history of congenital cytomegalovirus infection in a highly seroimmune population. Clin. Infect. Dis. 49, 522–528 10.1086/600882 19583520 PMC2778219

[34] Kenneson A. and Cannon M.J. (2007) Review and meta-analysis of the epidemiology of congenital cytomegalovirus (CMV) infection. Rev. Med. Virol. 17, 253–276 10.1002/rmv.535 17579921

[35] Turco M.Y. and Moffett A. (2019) Development of the human placenta. Development 146, dev163428 10.1242/dev.163428 31776138

[36] Ander S.E., Diamond M.S. and Coyne C.B. (2019) Immune responses at the maternal–fetal interface. Sci. Immunol. 4, eaat6114 10.1126/sciimmunol.aat6114 30635356 PMC6744611

[37] Malassine A., Frendo J.L. and Evain-Brion D. (2003) A comparison of placental development and endocrine functions between the human and mouse model. Hum. Reprod. Update 9, 531–539 10.1093/humupd/dmg043 14714590

[38] Njue A., Coyne C., Margulis A.V., Wang D., Marks M.A., Russell K. et al. (2020) The role of congenital cytomegalovirus infection in adverse birth outcomes: a review of the potential mechanisms. Viruses 13, 20 10.3390/v13010020 33374185 PMC7823935

[39] Hamilton W.J. and Boyd J.D. (1960) Development of the human placenta in the first three months of gestation. J. Anat. 94, 297–328 14399291 PMC1244370

[40] Boyd J.D. and Hamilton W.J. (1970) The Human Placenta, Heffer and Sons, Cambridge, U.K.

[41] Jauniaux E., Watson A.L., Hempstock J., Bao Y.P., Skepper J.N. and Burton G.J. (2000) Onset of maternal arterial blood flow and placental oxidative stress. A possible factor in human early pregnancy failure. Am. J. Pathol. 157, 2111–2122 10.1016/S0002-9440(10)64849-3 11106583 PMC1885754

[42] Burton G.J. and Jauniaux E. (2018) Development of the human placenta and fetal heart: synergic or independent? Front. Physiol. 9, 373 10.3389/fphys.2018.00373 29706899 PMC5906582

[43] Benirschke K., Burton G. and Baergen R. (2012) Pathology of the Human Placenta, 6th edn, Springer-Verlag, Berlin, 10.1007/978-3-642-23941-0

[44] Burton G.J. (2009) Oxygen, the Janus gas; its effects on human placental development andthi function. J. Anat. 215, 27–35 10.1111/j.1469-7580.2008.00978.x 19175804 PMC2714636

[45] Yoshida N., Appios A., Li Q., Hutton J.P., Wood G., Potts M. et al. (2025) Interactions between placental Hofbauer cells and L. monocytogenes change throughout gestation. Sci. Immunol. 10, eadq3066 10.1126/sciimmunol.adq3066 40680145

[46] Moffett-King A. (2002) Natural killer cells and pregnancy. Nat. Rev. Immunol. 2, 656–663 10.1038/nri886 12209134

[47] Williams P.J., Searle R.F., Robson S.C., Innes B.A. and Bulmer J.N. (2009) Decidual leucocyte populations in early to late gestation normal human pregnancy. J. Reprod. Immunol. 82, 24–31 10.1016/j.jri.2009.08.001 19732959

[48] Jabrane-Ferrat N. (2019) Features of human decidual NK Cells in healthy pregnancy and during viral infection. Front. Immunol. 10, 1397 10.3389/fimmu.2019.01397 31379803 PMC6660262

[49] Vento-Tormo R., Efremova M., Botting R.A., Turco M.Y., Vento-Tormo M., Meyer K.B. et al. (2018) Single-cell reconstruction of the early maternal–fetal interface in humans. Nature 563, 347–353 10.1038/s41586-018-0698-6 30429548 PMC7612850

[50] Huhn O., Ivarsson M.A., Gardner L., Hollinshead M., Stinchcombe J.C., Chen P. et al. (2020) Distinctive phenotypes and functions of innate lymphoid cells in human decidua during early pregnancy. Nat. Commun. 11, 381 10.1038/s41467-019-14123-z 31959757 PMC6971012

[51] Koopman L.A., Kopcow H.D., Rybalov B., Boyson J.E., Orange J.S., Schatz F. et al. (2003) Human decidual natural killer cells are a unique NK cell subset with immunomodulatory potential. J. Exp. Med. 198, 1201–1212 10.1084/jem.20030305 14568979 PMC2194228

[52] Li Q., Sharkey A., Sheridan M., Magistrati E., Arutyunyan A., Huhn O. et al. (2024) Human uterine natural killer cells regulate differentiation of extravillous trophoblast early in pregnancy. Cell Stem Cell 31, 181.e189–195.e189 10.1016/j.stem.2023.12.013 38237587

[53] Huhn O., Zhao X., Esposito L., Moffett A., Colucci F. and Sharkey A.M. (2021) How do uterine natural killer and innate lymphoid cells contribute to successful pregnancy? Front. Immunol. 12, 607669 10.3389/fimmu.2021.607669 34234770 PMC8256162

[54] Brown E., Martinez-Aguilar R., Maybin J.A. and Gibson D.A. (2022) Endometrial macrophages in health and disease. Int. Rev. Cell. Mol. Biol. 367, 183–208 10.1016/bs.ircmb.2022.03.011 35461658

[55] Houser B.L., Tilburgs T., Hill J., Nicotra M.L. and Strominger J.L. (2011) Two unique human decidual macrophage populations. J. Immunol. 186, 2633–2642 10.4049/jimmunol.1003153 21257965 PMC3712354

[56] Vondra S., Hobler A.L., Lackner A.I., Raffetseder J., Mihalic Z.N., Vogel A. et al. (2023) The human placenta shapes the phenotype of decidual macrophages. Cell Rep. 42, 112285 10.1016/j.celrep.2023.112285 36917611

[57] Nowakowski T.J., Pollen A.A., Di Lullo E., Sandoval-Espinosa C., Bershteyn M. and Kriegstein A.R. (2016) Expression analysis highlights AXL as a candidate Zika virus entry receptor in neural stem cells. Cell Stem Cell 18, 591–596 10.1016/j.stem.2016.03.012 27038591 PMC4860115

[58] Thomas J.R., Appios A., Zhao X., Dutkiewicz R., Donde M., Lee C.Y.C. et al. (2021) Phenotypic and functional characterization of first-trimester human placental macrophages, Hofbauer cells. J. Exp. Med. 218, e20200891 10.1084/jem.20200891 33075123 PMC7579740

[59] Pierleoni C., Castellucci M., Kaufmann P., Lund L.R. and Schnack Nielsen B. (2003) Urokinase receptor is up-regulated in endothelial cells and macrophages associated with fibrinoid deposits in the human placenta. Placenta 24, 677–685 10.1016/S0143-4004(03)00082-1 12828926

[60] Burton G.J. and Watson A.L. (1997) The structure of the human placenta: implications for initiating and defending against virus infections. Rev. Med. Virol. 7, 219–228 10.1002/(SICI)1099-1654(199712)7:4<219::AID-RMV205>3.0.CO;2-E 10398486

[61] Ramachandran P., Dobie R., Wilson-Kanamori J.R., Dora E.F., Henderson B.E.P., Luu N.T. et al. (2019) Resolving the fibrotic niche of human liver cirrhosis at single-cell level. Nature 575, 512–518 10.1038/s41586-019-1631-3 31597160 PMC6876711

[62] Zhu Y., Cai P., Li Z., Zhang S., Kong W.T., Wang H. et al. (2025) Transcription factors TCF4 and KLF4 respectively control the development of the DC2A and DC2B lineages. Nat. Immunol. 26, 1275–1286 10.1038/s41590-025-02208-5 40702338

[63] Bigley V. and Collin M. (2025) 2B or not 2B is the question in DC ontogeny. Nat. Immunol. 26, 1214–1216 10.1038/s41590-025-02220-9 40702339

[64] Bachem A., Guttler S., Hartung E., Ebstein F., Schaefer M., Tannert A. et al. (2010) Superior antigen cross-presentation and XCR1 expression define human CD11c ^+^ CD141^+^ cells as homologues of mouse CD8^+^ dendritic cells. J. Exp. Med. 207, 1273–1281 10.1084/jem.20100348 20479115 PMC2882837

[65] Wei R., Lai N., Zhao L., Zhang Z., Zhu X., Guo Q. et al. (2021) Dendritic cells in pregnancy and pregnancy-associated diseases. Biomed. Pharmacother. 133, 110921 10.1016/j.biopha.2020.110921 33378991

[66] Ginhoux F., Guilliams M. and Merad M. (2022) Expanding dendritic cell nomenclature in the single-cell era. Nat. Rev. Immunol. 22, 67–68 10.1038/s41577-022-00675-7 35027741

[67] Volchek M., Girling J.E., Lash G.E., Cann L., Kumar B., Robson S.C. et al. (2010) Lymphatics in the human endometrium disappear during decidualization. Hum. Reprod. 25, 2455–2464 10.1093/humrep/deq224 20729537

[68] Tilburgs T. and Strominger J.L. (2013) CD8+ effector T cells at the fetal-maternal interface, balancing fetal tolerance and antiviral immunity. Am. J. Reprod. Immunol. 69, 395–407 10.1111/aji.12094 23432707 PMC3711858

[69] Tilburgs T., Claas F.H. and Scherjon S.A. (2010) Elsevier Trophoblast Research Award Lecture: unique properties of decidual T cells and their role in immune regulation during human pregnancy.. Placenta 31 Suppl, S82–S86 10.1016/j.placenta.2010.01.007 20106522

[70] Moffett A. and Shreeve N. (2023) Local immune recognition of trophoblast in early human pregnancy: controversies and questions. Nat. Rev. Immunol. 23, 222–235 10.1038/s41577-022-00777-2 36192648 PMC9527719

[71] Wegmann T.G., Lin H., Guilbert L. and Mosmann T.R. (1993) Bidirectional cytokine interactions in the maternal–fetal relationship: is successful pregnancy a TH2 phenomenon? Immunol. Today 14, 353–356 10.1016/0167-5699(93)90235-D 8363725

[72] Sykes L., MacIntyre D.A., Yap X.J., Teoh T.G. and Bennett P.R. (2012) The Th1:th2 dichotomy of pregnancy and preterm labour. Mediators Inflamm. 2012, 967629 10.1155/2012/967629 22719180 PMC3376783

[73] Powell R.M., Lissauer D., Tamblyn J., Beggs A., Cox P., Moss P. et al. (2017) Decidual T cells exhibit a highly differentiated phenotype and demonstrate potential fetal specificity and a strong transcriptional response to IFN. J. Immunol. 199, 3406–3417 10.4049/jimmunol.1700114 28986438 PMC5679367

[74] Tilburgs T., Schonkeren D., Eikmans M., Nagtzaam N.M., Datema G., Swings G.M. et al. (2010) Human decidual tissue contains differentiated CD8^+^ effector-memory T cells with unique properties. J. Immunol. 185, 4470–4477 10.4049/jimmunol.0903597 20817873

[75] van der Zwan A., Bi K., Norwitz E.R., Crespo A.C., Claas F.H.J., Strominger J.L. et al. (2018) Mixed signature of activation and dysfunction allows human decidual CD8^+^ T cells to provide both tolerance and immunity. Proc. Natl. Acad. Sci. U.S.A. 115, 385–390 10.1073/pnas.1713957115 29259116 PMC5777048

[76] Fisher S., Genbacev O., Maidji E. and Pereira L. (2000) Human cytomegalovirus infection of placental cytotrophoblasts *in vitro* and *in utero*: implications for transmission and pathogenesis. J. Virol. 74, 6808–6820 10.1128/JVI.74.15.6808-6820.2000 10888620 PMC112198

[77] Pereira L., Maidji E., McDonagh S., Genbacev O. and Fisher S. (2003) Human cytomegalovirus transmission from the uterus to the placenta correlates with the presence of pathogenic bacteria and maternal immunity. J. Virol. 77, 13301–13314 10.1128/JVI.77.24.13301-13314.2003 14645586 PMC296088

[78] Smith M.S., Bentz G.L., Alexander J.S. and Yurochko A.D. (2004) Human cytomegalovirus induces monocyte differentiation and migration as a strategy for dissemination and persistence. J. Virol. 78, 4444–4453 10.1128/JVI.78.9.4444-4453.2004 15078925 PMC387677

[79] Sinclair J. and Poole E. (2014) Human cytomegalovirus latency and reactivation in and beyond the myeloid lineage. Future Virology 9, 557–563 10.2217/fvl.14.34 18164651

[80] Poole E. and Sinclair J. (2015) Sleepless latency of human cytomegalovirus. Med. Microbiol. Immunol. 204, 421–429 10.1007/s00430-015-0401-6 25772624 PMC4439429

[81] Muhlemann K., Miller R.K., Metlay L. and Menegus M.A. (1992) Cytomegalovirus infection of the human placenta: an immunocytochemical study. Hum. Pathol. 23, 1234–1237 10.1016/0046-8177(92)90290-J 1330874

[82] Fock V., Plessl K., Fuchs R., Dekan S., Milla S.K., Haider S. et al. (2015) Trophoblast subtype-specific EGFR/ERBB4 expression correlates with cell cycle progression and hyperplasia in complete hydatidiform moles. Hum. Reprod. 30, 789–799 10.1093/humrep/dev027 25740878

[83] Maidji E., McDonagh S., Genbacev O., Tabata T. and Pereira L. (2006) Maternal antibodies enhance or prevent cytomegalovirus infection in the placenta by neonatal Fc receptor-mediated transcytosis. Am. J. Pathol. 168, 1210–1226 10.2353/ajpath.2006.050482 16565496 PMC1606573

[84] Ander S.E., Rudzki E.N., Arora N., Sadovsky Y., Coyne C.B. and Boyle J.P. (2018) Human placental syncytiotrophoblasts restrict *Toxoplasma gondii* attachment and replication and respond to infection by producing immunomodulatory chemokines. mBio 9, e01678–17 10.1128/mBio.01678-17 29317509 PMC5760739

[85] Delorme-Axford E., Donker R.B., Mouillet J.F., Chu T., Bayer A., Ouyang Y. et al. (2013) Human placental trophoblasts confer viral resistance to recipient cells. Proc. Natl. Acad. Sci. U.S.A. 110, 12048–12053 10.1073/pnas.1304718110 23818581 PMC3718097

[86] Bayer A., Lennemann N.J., Ouyang Y., Bramley J.C., Morosky S., Marques E.T. Jr. et al. (2016) Type III interferons produced by human placental trophoblasts confer protection against zika virus infection. Cell Host Microbe 19, 705–712 10.1016/j.chom.2016.03.008 27066743 PMC4866896

[87] Jagger B.W., Miner J.J., Cao B., Arora N., Smith A.M., Kovacs A. et al. (2017) Gestational stage and IFN-lambda signaling regulate ZIKV infection *in utero*. Cell Host Microbe 22, 366.e363–376.e363 10.1016/j.chom.2017.08.012 28910635 PMC5647680

[88] Hemmings D.G. and Guilbert L.J. (2002) Polarized release of human cytomegalovirus from placental trophoblasts. J. Virol. 76, 6710–6717 10.1128/JVI.76.13.6710-6717.2002 12050384 PMC136275

[89] Maidji E., Percivalle E., Gerna G., Fisher S. and Pereira L. (2002) Transmission of human cytomegalovirus from infected uterine microvascular endothelial cells to differentiating/invasive placental cytotrophoblasts. Virology 304, 53–69 10.1006/viro.2002.1661 12490403

[90] Weisblum Y., Panet A., Zakay-Rones Z., Haimov-Kochman R., Goldman-Wohl D., Ariel I. et al. (2011) Modeling of human cytomegalovirus maternal–fetal transmission in a novel decidual organ culture. J. Virol. 85, 13204–13213 10.1128/JVI.05749-11 21976654 PMC3233115

[91] Wang X., Huang D.Y., Huong S.M. and Huang E.S. (2005) Integrin alphavbeta3 is a coreceptor for human cytomegalovirus. Nat. Med. 11, 515–521 10.1038/nm1236 15834425 PMC1904494

[92] Maidji E., Genbacev O., Chang H.T. and Pereira L. (2007) Developmental regulation of human cytomegalovirus receptors in cytotrophoblasts correlates with distinct replication sites in the placenta. J. Virol. 81, 4701–4712 10.1128/JVI.02748-06 17314173 PMC1900158

[93] Compton T. (2004) Receptors and immune sensors: the complex entry path of human cytomegalovirus. Trends Cell Biol. 14, 5–8 10.1016/j.tcb.2003.10.009 14729174

[94] Wu H., Huang X.Y., Sun M.X., Wang Y., Zhou H.Y., Tian Y. et al. (2023) Zika virus targets human trophoblast stem cells and prevents syncytialization in placental trophoblast organoids. Nat. Commun. 14, 5541 10.1038/s41467-023-41158-0 37684223 PMC10491779

[95] Tabata T., McDonagh S., Kawakatsu H. and Pereira L. (2007) Cytotrophoblasts infected with a pathogenic human cytomegalovirus strain dysregulate cell-matrix and cell-cell adhesion molecules: a quantitative analysis. Placenta 28, 527–537 10.1016/j.placenta.2006.05.006 16822542

[96] Krstanovic F., Britt W.J., Jonjic S. and Brizic I. (2021) Cytomegalovirus infection and inflammation in developing brain. Viruses 13, 1078 10.3390/v13061078 34200083 PMC8227981

[97] Gabrielli L., Bonasoni M.P., Santini D., Piccirilli G., Chiereghin A., Guerra B. et al. (2013) Human fetal inner ear involvement in congenital cytomegalovirus infection. Acta Neuropathol. Commun. 1, 63 10.1186/2051-5960-1-63 24252374 PMC3893406

[98] Preston H., Casey R., Ferris E., Kerr-Jones L., Jones L., Latif F. et al. (2025) Human cytomegalovirus immune evasion of natural killer cells: a virus for all seasons? Pathogens 14, 629 10.3390/pathogens14070629 40732677 PMC12300392

[99] Manaster I. and Mandelboim O. (2010) The unique properties of uterine NK cells. Am. J. Reprod. Immunol. 63, 434–444 10.1111/j.1600-0897.2009.00794.x 20055791

[100] Siewiera J., El Costa H., Tabiasco J., Berrebi A., Cartron G., Le Bouteiller P. et al. (2013) Human cytomegalovirus infection elicits new decidual natural killer cell effector functions. PLoS Pathog. 9, e1003257 10.1371/journal.ppat.1003257 23592985 PMC3617138

[101] de Mendonca Vieira R., Meagher A., Crespo A.C., Kshirsagar S.K., Iyer V., Norwitz E.R. et al. (2020) Human term pregnancy decidual NK cells generate distinct cytotoxic responses. J. Immunol. 204, 3149–3159 10.4049/jimmunol.1901435 32376646 PMC7322730

[102] Crespo A.C., Strominger J.L. and Tilburgs T. (2016) Expression of KIR2DS1 by decidual natural killer cells increases their ability to control placental HCMV infection. Proc. Natl. Acad. Sci. U.S.A. 113, 15072–15077 10.1073/pnas.1617927114 27956621 PMC5206558

[103] Ljunggren H.G. and Karre K. (1990) In search of the ‘missing self’: MHC molecules and NK cell recognition. Immunol. Today 11, 237–244 10.1016/0167-5699(90)90097-S 2201309

[104] Lopez-Verges S., Milush J.M., Schwartz B.S., Pando M.J., Jarjoura J., York V.A. et al. (2011) Expansion of a unique CD57(+)NKG2Chi natural killer cell subset during acute human cytomegalovirus infection. Proc. Natl. Acad. Sci. U.S.A. 108, 14725–14732 10.1073/pnas.1110900108 21825173 PMC3169160

[105] Lissauer D., Choudhary M., Pachnio A., Goodyear O., Moss P.A. and Kilby M.D. (2011) Cytomegalovirus sero positivity dramatically alters the maternal CD8+ T cell repertoire and leads to the accumulation of highly differentiated memory cells during human pregnancy. Hum. Reprod. 26, 3355–3365 10.1093/humrep/der327 21979962

[106] Alfi O., Cohen M., Bar-On S., Hashimshony T., Levitt L., Raz Y. et al. (2024) Decidual-tissue-resident memory T cells protect against nonprimary human cytomegalovirus infection at the maternal–fetal interface. Cell Rep. 43, 113698 10.1016/j.celrep.2024.113698 38265934

[107] Tabata T., Petitt M., Fang-Hoover J. and Pereira L. (2019) Survey of cellular immune responses to human cytomegalovirus infection in the microenvironment of the uterine-placental interface. Med. Microbiol. Immunol. 208, 475–485 10.1007/s00430-019-00613-w 31065796 PMC6635015

[108] Kok W.L., Denney L., Benam K., Cole S., Clelland C., McMichael A.J. et al. (2012) Pivotal advance: Invariant NKT cells reduce accumulation of inflammatory monocytes in the lungs and decrease immune-pathology during severe influenza A virus infection. J. Leukoc. Biol. 91, 357–368 10.1189/jlb.0411184 22003207

[109] Zhang Y.H., He M., Wang Y. and Liao A.H. (2017) Modulators of the balance between M1 and M2 macrophages during pregnancy. Front. Immunol. 8, 120 10.3389/fimmu.2017.00120 28232836 PMC5299000

[110] Schwartz D.A., Khan R. and Stoll B. (1992) Characterization of the fetal inflammatory response to cytomegalovirus placentitis. An immunohistochemical study. Arch. Pathol. Lab. Med. 116, 21–27 1310378

[111] Euscher E., Davis J., Holzman I. and Nuovo G.J. (2001) Coxsackie virus infection of the placenta associated with neurodevelopmental delays in the newborn. Obstet. Gynecol. 98, 1019–1026 11755547 10.1016/s0029-7844(01)01625-8

[112] Satosar A., Ramirez N.C., Bartholomew D., Davis J. and Nuovo G.J. (2004) Histologic correlates of viral and bacterial infection of the placenta associated with severe morbidity and mortality in the newborn. Hum. Pathol. 35, 536–545 10.1016/j.humpath.2004.01.015 15138926

[113] Faye-Petersen O.M. (2008) The placenta in preterm birth. J. Clin. Pathol. 61, 1261–1275 10.1136/jcp.2008.055244 19074631

[114] Johnson E.L., Boggavarapu S., Johnson E.S., Lal A.A., Agrawal P., Bhaumik S.K. et al. (2018) Human cytomegalovirus enhances placental susceptibility and replication of human immunodeficiency virus type 1 (HIV-1), which may facilitate *in utero* HIV-1 transmission. J. Infect. Dis. 218, 1464–1473 10.1093/infdis/jiy327 29860306 PMC6927849

[115] Wussow F., Chiuppesi F., Martinez J., Campo J., Johnson E., Flechsig C. et al. (2014) Human cytomegalovirus vaccine based on the envelope gH/gL pentamer complex. PLoS Pathog. 10, e1004524 10.1371/journal.ppat.1004524 25412505 PMC4239111

[116] Bayer A., Delorme-Axford E., Sleigher C., Frey T.K., Trobaugh D.W., Klimstra W.B. et al. (2015) Human trophoblasts confer resistance to viruses implicated in perinatal infection. Am. J. Obstet. Gynecol. 212, 71.e71–71.e78 10.1016/j.ajog.2014.07.060 PMC4275367 25108145

[117] Pudney J., He X., Masheeb Z., Kindelberger D.W., Kuohung W. and Ingalls R.R. (2016) Differential expression of toll-like receptors in the human placenta across early gestation. Placenta 46, 1–10 10.1016/j.placenta.2016.07.005 27697215 PMC5119647

[118] Dhar R., Singh S., Sahoo O.S., Chandra N., Gul A., Mukherjee I. et al. (2025) The placental battlefield: viral strategies and immune countermeasures. Front. Immunol. 16, 1667601 10.3389/fimmu.2025.1667601 41479918 PMC12754011

[119] Semmes E.C. and Coyne C.B. (2022) Innate immune defenses at the maternal–fetal interface. Curr. Opin. Immunol. 74, 60–67 10.1016/j.coi.2021.10.007 34768027 PMC11063961

[120] Olmos-Ortiz A., Flores-Espinosa P., Mancilla-Herrera I., Vega-Sanchez R., Diaz L. and Zaga-Clavellina V. (2019) Innate immune cells and toll-like receptor-dependent responses at the maternal–fetal interface. Int. J. Mol. Sci. 20, 3654 10.3390/ijms20153654 31357391 PMC6695670

[121] Bulmer J.N., Longfellow M. and Ritson A. (1991) Leukocytes and resident blood cells in endometrium. Ann. N. Y. Acad. Sci. 622, 57–68 10.1111/j.1749-6632.1991.tb37850.x 2064208

[122] Vacca P., Montaldo E., Croxatto D., Loiacono F., Canegallo F., Venturini P.L. et al. (2015) Identification of diverse innate lymphoid cells in human decidua. Mucosal. Immunol. 8, 254–264 10.1038/mi.2014.63 25052762

[123] Gibbs A., Leeansyah E., Introini A., Paquin-Proulx D., Hasselrot K., Andersson E. et al. (2017) MAIT cells reside in the female genital mucosa and are biased towards IL-17 and IL-22 production in response to bacterial stimulation. Mucosal. Immunol. 10, 35–45 10.1038/mi.2016.30 27049062 PMC5053908

[124] Darmochwal-Kolarz D.A., Kludka-Sternik M., Chmielewski T., Kolarz B., Rolinski J., Leszczynska-Gorzelak B. et al. (2012) The expressions of CD200 and CD200R molecules on myeloid and lymphoid dendritic cells in pre-eclampsia and normal pregnancy. Am. J. Reprod. Immunol. 67, 474–481 10.1111/j.1600-0897.2012.01126.x 22462561

[125] Aronoff D.M., Correa H., Rogers L.M., Arav-Boger R. and Alcendor D.J. (2017) Placental pericytes and cytomegalovirus infectivity: Implications for HCMV placental pathology and congenital disease. Am. J. Reprod. Immunol. 78, 10.1111/aji.12728 28741727 PMC5561471

[126] Mi S., Lee X., Li X., Veldman G.M., Finnerty H., Racie L. et al. (2000) Syncytin is a captive retroviral envelope protein involved in human placental morphogenesis. Nature 403, 785–789 10.1038/35001608 10693809

[127] Amsler L., Verweij M. and DeFilippis V.R. (2013) The tiers and dimensions of evasion of the type I interferon response by human cytomegalovirus. J. Mol. Biol. 425, 4857–4871 10.1016/j.jmb.2013.08.023 24013068 PMC3864659

[128] Lee J.K., Kim J.E., Park B.J. and Song Y.J. (2020) Human cytomegalovirus IE86 protein aa 136-289 mediates STING degradation and blocks the cGAS-STING pathway. J. Microbiol. 58, 54–60 10.1007/s12275-020-9577-6 31898253

[129] Taylor R.T. and Bresnahan W.A. (2006) Human cytomegalovirus immediate-early 2 protein IE86 blocks virus-induced chemokine expression. J. Virol. 80, 920–928 10.1128/JVI.80.2.920-928.2006 16378994 PMC1346867

[130] Poole E., Atkins E., Nakayama T., Yoshie O., Groves I., Alcami A. et al. (2008) NF-kappaB-mediated activation of the chemokine CCL22 by the product of the human cytomegalovirus gene UL144 escapes regulation by viral IE86. J. Virol. 82, 4250–4256 10.1128/JVI.02156-07 18287226 PMC2293074

[131] Choi H.J., Park A., Kang S., Lee E., Lee T.A., Ra E.A. et al. (2018) Human cytomegalovirus-encoded US9 targets MAVS and STING signaling to evade type I interferon immune responses. Nat. Commun. 9, 125 10.1038/s41467-017-02624-8 29317664 PMC5760629

[132] Kim Y.J., Kim E.T., Kim Y.E., Lee M.K., Kwon K.M., Kim K.I. et al. (2016) Consecutive inhibition of ISG15 expression and ISGylation by cytomegalovirus regulators. PLoS Pathog. 12, e1005850 10.1371/journal.ppat.1005850 27564865 PMC5001722

[133] Weisblum Y., Oiknine-Djian E., Zakay-Rones Z., Vorontsov O., Haimov-Kochman R., Nevo Y. et al. (2017) APOBEC3A is upregulated by human cytomegalovirus (HCMV) in the maternal–fetal interface, acting as an innate Anti-HCMV effector. J. Virol. 91, e01296–17 10.1128/JVI.01296-17 28956761 PMC5686750

[134] Pautasso S., Galitska G., Dell'Oste V., Biolatti M., Cagliani R., Forni D. et al. (2018) Strategy of human cytomegalovirus to escape interferon beta-induced APOBEC3G editing activity. J. Virol. 92, e01224–18 10.1128/JVI.01224-18 30045985 PMC6146821

[135] Weisblum Y., Panet A., Zakay-Rones Z., Vitenshtein A., Haimov-Kochman R., Goldman-Wohl D. et al. (2015) Human cytomegalovirus induces a distinct innate immune response in the maternal–fetal interface. Virology 485, 289–296 10.1016/j.virol.2015.06.023 26318261

[136] Scott G.M., Chow S.S., Craig M.E., Pang C.N., Hall B., Wilkins M.R. et al. (2012) Cytomegalovirus infection during pregnancy with maternofetal transmission induces a proinflammatory cytokine bias in placenta and amniotic fluid. J. Infect. Dis. 205, 1305–1310 10.1093/infdis/jis186 22383678

[137] Tabata T., Kawakatsu H., Maidji E., Sakai T., Sakai K., Fang-Hoover J. et al. (2008) Induction of an epithelial integrin alphavbeta6 in human cytomegalovirus-infected endothelial cells leads to activation of transforming growth factor-beta1 and increased collagen production. Am. J. Pathol. 172, 1127–1140 10.2353/ajpath.2008.070448 18349127 PMC2276431

[138] Jackson S.E., Mason G.M. and Wills M.R. (2011) Human cytomegalovirus immunity and immune evasion. Virus Res. 157, 151–160 10.1016/j.virusres.2010.10.031 21056604

[139] Terauchi M., Koi H., Hayano C., Toyama-Sorimachi N., Karasuyama H., Yamanashi Y. et al. (2003) Placental extravillous cytotrophoblasts persistently express class I major histocompatibility complex molecules after human cytomegalovirus infection. J. Virol. 77, 8187–8195 10.1128/JVI.77.15.8187-8195.2003 12857887 PMC165235

[140] King A., Hiby S.E., Gardner L., Joseph S., Bowen J.M., Verma S. et al. (2000) Recognition of trophoblast HLA class I molecules by decidual NK cell receptors—a review. Placenta 21 Suppl A, S81–S85 10.1053/plac.1999.0520 10831129

[141] Tomasec P., Braud V.M., Rickards C., Powell M.B., McSharry B.P., Gadola S. et al. (2000) Surface expression of HLA-E, an inhibitor of natural killer cells, enhanced by human cytomegalovirus gpUL40. Science 287, 1031 10.1126/science.287.5455.1031 10669413

[142] Tarrago D., Gonzalez I. and Gonzalez-Escribano M.F. (2022) HLA-E restricted cytomegalovirus UL40 peptide polymorphism may represent a risk factor following congenital infection. BMC Genomics 23, 455 10.1186/s12864-022-08689-0 35725386 PMC9208114

[143] Hammer Q., Ruckert T., Borst E.M., Dunst J., Haubner A., Durek P. et al. (2018) Peptide-specific recognition of human cytomegalovirus strains controls adaptive natural killer cells. Nat. Immunol. 19, 453–463 10.1038/s41590-018-0082-6 29632329

[144] Prod'homme V., Griffin C., Aicheler R.J., Wang E.C., McSharry B.P., Rickards C.R. et al. (2007) The human cytomegalovirus MHC class I homolog UL18 inhibits LIR-1+ but activates LIR-1- NK cells. J. Immunol. 178, 4473–4481 10.4049/jimmunol.178.7.4473 17372005 PMC2843079

[145] Xu X., Zhou Y. and Wei H. (2020) Roles of HLA-G in the maternal–fetal immune microenvironment. Front. Immunol. 11, 592010 10.3389/fimmu.2020.592010 33193435 PMC7642459

[146] Reyburn H.T., Mandelboim O., Vales-Gomez M., Davis D.M., Pazmany L. and Strominger J.L. (1997) The class I MHC homologue of human cytomegalovirus inhibits attack by natural killer cells. Nature 386, 514–517 10.1038/386514a0 9087413

[147] Leon-Juarez M., Martinez-Castillo M., Gonzalez-Garcia L.D., Helguera-Repetto A.C., Zaga-Clavellina V., Garcia-Cordero J. et al. (2017) Cellular and molecular mechanisms of viral infection in the human placenta. Pathog. Dis. 75, ftx093 10.1093/femspd/ftx093 28903546 PMC7108519

[148] McDonagh S., Maidji E., Chang H.T. and Pereira L. (2006) Patterns of human cytomegalovirus infection in term placentas: a preliminary analysis. J. Clin. Virol. 35, 210–215 10.1016/j.jcv.2005.08.011 16386950

[149] Adler S.P. (2011) Screening for cytomegalovirus during pregnancy. Infect. Dis. Obstet. Gynecol. 2011, 1–9 10.1155/2011/942937 21836812 PMC3152970

[150] Beaudoin M.L., Renaud C., Boucher M., Kakkar F., Gantt S. and Boucoiran I. (2021) Perspectives of women on screening and prevention of CMV in pregnancy. Eur. J. Obstet. Gynecol. Reprod. Biol. 258, 409–413 10.1016/j.ejogrb.2021.01.035 33548895

[151] Sanchez-Duran M.A., Maiz N., Liutsko L., Bielsa-Pascual J., Garcia-Sierra R., Zientalska A.M. et al. (2023) Universal screening programme for cytomegalovirus infection in the first trimester of pregnancy: study protocol for an observational multicentre study in the area of Barcelona (CITEMB study). BMJ Open 13, e071997 10.1136/bmjopen-2023-071997 37474185 PMC10357649

[152] Leruez-Ville M., Chatzakis C., Lilleri D., Blazquez-Gamero D., Alarcon A., Bourgon N. et al. (2024) Consensus recommendation for prenatal, neonatal and postnatal management of congenital cytomegalovirus infection from the European congenital infection initiative (ECCI). Lancet Reg. Health Eur. 40, 100892 10.1016/j.lanepe.2024.100892 38590940 PMC10999471

[153] Mace M., Sissoeff L., Rudent A. and Grangeot-Keros L. (2004) A serological testing algorithm for the diagnosis of primary CMV infection in pregnant women. Prenat. Diagn. 24, 861–863 10.1002/pd.1001 15565653

[154] Weimer K.E.D. and Permar S.R. (2019) Chapter 3 - When and How to Treat Neonatal CMV Infection. In Infectious Disease and Pharmacology, pp. 27–36, Elsevier, Philadelphia

[155] Picone O., Grangeot-Keros L., Senat M., Fuchs F., Bouthry E., Ayoubi J. et al. (2017) Cytomegalovirus non-primary infection during pregnancy. Can serology help with diagnosis? J. Matern. Fetal Neonatal Med. 30, 224–227 10.3109/14767058.2016.1169521 27147102

[156] Salome S., Corrado F.R., Mazzarelli L.L., Maruotti G.M., Capasso L., Blazquez-Gamero D. et al. (2023) Congenital cytomegalovirus infection: the state of the art and future perspectives. Front. Pediatr. 11, 1276912 10.3389/fped.2023.1276912 38034830 PMC10687293

[157] Guerra B., Simonazzi G., Puccetti C., Lanari M., Farina A., Lazzarotto T. et al. (2008) Ultrasound prediction of symptomatic congenital cytomegalovirus infection. Am. J. Obstet. Gynecol. 198, 380.e381–380.e387 10.1016/j.ajog.2007.09.052 18191802

[158] Bourgon N., Fourgeaud J., Daclin C., Loeuillet L., Bessieres B., Faure-Bardon V. et al. (2025) Early prediction of congenital cytomegalovirus infection and symptoms after maternal primary infection: an *in vivo* study using CMV-PCR in chorionic villi and in amniotic fluid. Am. J. Obstet. Gynecol. 234, 1130–1147 10.1016/j.ajog.2025.11.018 41265737

[159] Arvin A.M., Fast P., Myers M., Plotkin S., Rabinovich R.and National Vaccine Advisory, C (2004) Vaccine development to prevent cytomegalovirus disease: report from the National Vaccine Advisory Committee. Clin. Infect. Dis. 39, 233–239 10.1086/421999 15307033

[160] Pass R.F., Zhang C., Evans A., Simpson T., Andrews W., Huang M.L. et al. (2009) Vaccine prevention of maternal cytomegalovirus infection. N. Engl. J. Med. 360, 1191–1199 10.1056/NEJMoa0804749 19297572 PMC2753425

[161] Bernstein D.I., Munoz F.M., Callahan S.T., Rupp R., Wootton S.H., Edwards K.M. et al. (2016) Safety and efficacy of a cytomegalovirus glycoprotein B (gB) vaccine in adolescent girls: a randomized clinical trial. Vaccine 34, 313–319 10.1016/j.vaccine.2015.11.056 26657184 PMC4701617

[162] Hu X., Karthigeyan K.P., Herbek S., Valencia S.M., Jenks J.A., Webster H. et al. (2024) Human cytomegalovirus mRNA-1647 vaccine candidate elicits potent and broad neutralization and higher antibody-dependent cellular cytotoxicity responses than the gB/MF59 vaccine. J. Infect. Dis. 230, 455–466 10.1093/infdis/jiad593 38324766 PMC11326847

[163] Permar S.R., Schleiss M.R. and Plotkin S.A. (2025) A vaccine against cytomegalovirus: how close are we? J. Clin. Invest. 135, e182317 10.1172/JCI182317 39744948 PMC11684802

[164] D'Antonio F., Marinceu D., Prasad S. and Khalil A. (2023) Effectiveness and safety of prenatal valacyclovir for congenital cytomegalovirus infection: systematic review and meta-analysis. Ultrasound Obstet. Gynecol. 61, 436–444 10.1002/uog.26136 36484439

[165] Leruez-Ville M., Chatzakis C., Lilleri D., Blazquez-Gamero D., Alarcon A., Bourgon N. et al. (2024) Corrigendum to “Consensus recommendation for prenatal, neonatal and postnatal management of congenital cytomegalovirus infection from the European congenital infection initiative (ECCI)” [The Lancet Regional Health - Europe 40 (2024) 100892]. Lancet Reg. Health Eur. 42, 100974 10.1016/j.lanepe.2024.100974 38590940 PMC10999471

[166] Khalil A., Heath P.T., Jones C.E., Soe A., Ville Y.G. and Royal College of, O. and Gynaecologists (2025) Congenital cytomegalovirus infection: update on screening, diagnosis and treatment: Scientific Impact Paper No. 56. BJOG 132, e42–e52 10.1111/1471-0528.17966 39434207

[167] Gourin C., Alain S. and Hantz S. (2023) Anti-CMV therapy, what next? A systematic review Front. Microbiol. 14, 1321116 10.3389/fmicb.2023.1321116 38053548 PMC10694278

[168] Borst E.M., Hahn G., Koszinowski U.H. and Messerle M. (1999) Cloning of the human cytomegalovirus (HCMV) genome as an infectious bacterial artificial chromosome in *Escherichia coli*: a new approach for construction of HCMV mutants. J. Virol. 73, 8320–8329 10.1128/JVI.73.10.8320-8329.1999 10482582 PMC112849

[169] Stanton R.J., Baluchova K., Dargan D.J., Cunningham C., Sheehy O., Seirafian S. et al. (2010) Reconstruction of the complete human cytomegalovirus genome in a BAC reveals RL13 to be a potent inhibitor of replication. J. Clin. Invest. 120, 3191–3208 10.1172/JCI42955 20679731 PMC2929729

[170] Wilkinson G.W., Davison A.J., Tomasec P., Fielding C.A., Aicheler R., Murrell I. et al. (2015) Human cytomegalovirus: taking the strain. Med. Microbiol. Immunol. 204, 273–284 10.1007/s00430-015-0411-4 25894764 PMC4439430

[171] Furukawa S., Kuroda Y. and Sugiyama A. (2014) A comparison of the histological structure of the placenta in experimental animals. J. Toxicol. Pathol. 27, 11–18 10.1293/tox.2013-0060 24791062 PMC4000068

[172] Enders A.C. and Blankenship T.N. (1999) Comparative placental structure. Adv. Drug. Deliv. Rev. 38, 3–15 10.1016/S0169-409X(99)00003-4 10837743

[173] Manuel T.D., Mostrom M.J., Crooks C.M., Davalos A., Barfield R., Scheef E.A. et al. (2025) A rhesus macaque model of congenital cytomegalovirus infection reveals a spectrum of vertical transmission outcomes. Commun. Biol. 8, 1647 10.1038/s42003-025-09033-4 41286406 PMC12644875

[174] Li R.Y. and Tsutsui Y. (2000) Growth retardation and microcephaly induced in mice by placental infection with murine cytomegalovirus. Teratology 62, 79–85 10.1002/1096-9926(200008)62:2<79::AID-TERA3>3.0.CO;2-S 10931504

[175] Kawasaki H., Kosugi I., Arai Y. and Tsutsui Y. (2002) The amount of immature glial cells in organotypic brain slices determines the susceptibility to murine cytomegalovirus infection. Lab. Invest. 82, 1347–1358 10.1097/01.LAB.0000032376.58688.D4 12379769

[176] Johnson K.P. (1969) Mouse cytomegalovirus: placental infection. J. Infect. Dis. 120, 445–450 10.1093/infdis/120.4.445 4309994

[177] Forbes C.A., Brown M.G., Cho R., Shellam G.R., Yokoyama W.M. and Scalzo A.A. (1997) The Cmv1 host resistance locus is closely linked to the Ly49 multigene family within the natural killer cell gene complex on mouse chromosome 6. Genomics 41, 406–413 10.1006/geno.1997.4667 9169139

[178] Rodriguez M., Sabastian P., Clark P. and Brown M.G. (2004) Cmv1-independent antiviral role of NK cells revealed in murine cytomegalovirus-infected New Zealand White mice. J. Immunol. 173, 6312–6318 10.4049/jimmunol.173.10.6312 15528370

[179] Choi Y.C. and Hsiung G.D. (1978) Cytomegalovirus infection in guinea pigs. II. Transplacental and horizontal transmission. J. Infect. Dis. 138, 197–202 10.1093/infdis/138.2.197 210239

[180] Griffith B.P., McCormick S.R., Fong C.K., Lavallee J.T., Lucia H.L. and Goff E. (1985) The placenta as a site of cytomegalovirus infection in guinea pigs. J. Virol. 55, 402–409 10.1128/jvi.55.2.402-409.1985 2991565 PMC254947

[181] Johnson K.P. and Connor W.S. (1979) Guinea pig cytomegalovirus: transplacental transmission. Brief report. Arch. Virol. 59, 263–267 10.1007/BF01317422 222240

[182] Tarantal A.F., Salamat M.S., Britt W.J., Luciw P.A., Hendrickx A.G. and Barry P.A. (1998) Neuropathogenesis induced by rhesus cytomegalovirus in fetal rhesus monkeys (*Macaca mulatta*). J. Infect. Dis. 177, 446–450 10.1086/514206 9466534

[183] Barry P.A., Lockridge K.M., Salamat S., Tinling S.P., Yue Y., Zhou S.S. et al. (2006) Nonhuman primate models of intrauterine cytomegalovirus infection. ILAR J. 47, 49–64 10.1093/ilar.47.1.49 16391431

[184] Faye A., Pornprasert S., Dolcini G., Ave P., Taieb J., Taupin J.L. et al. (2005) Evaluation of the placental environment with a new *in vitro* model of histocultures of early and term placentae: determination of cytokine and chemokine expression profiles. Placenta 26, 262–267 10.1016/j.placenta.2004.08.005 15708128

[185] Amirhessami-Aghili N., Manalo P., Hall M.R., Tibbitts F.D., Ort C.A. and Afsari A. (1987) Human cytomegalovirus infection of human placental explants in culture: histologic and immunohistochemical studies. Am. J. Obstet. Gynecol. 156, 1365–1374 10.1016/0002-9378(87)90002-0 3035925

[186] Gabrielli L., Losi L., Varani S., Lazzarotto T., Eusebi V. and Landini M.P. (2001) Complete replication of human cytomegalovirus in explants of first trimester human placenta. J. Med. Virol. 64, 499–504 10.1002/jmv.1077 11468735

[187] Lopez H., Benard M., Saint-Aubert E., Baron M., Martin H., Al Saati T. et al. (2011) Novel model of placental tissue explants infected by cytomegalovirus reveals different permissiveness in early and term placentae and inhibition of indoleamine 2,3-dioxygenase activity. Placenta 32, 522–530 10.1016/j.placenta.2011.04.016 21605903

[188] Benard M., Straat K., Omarsdottir S., Leghmari K., Bertrand J., Davrinche C. et al. (2014) Human cytomegalovirus infection induces leukotriene B4 and 5-lipoxygenase expression in human placentae and umbilical vein endothelial cells. Placenta 35, 345–350 10.1016/j.placenta.2014.03.022 24746852

[189] Hamilton S.T., Scott G., Naing Z., Iwasenko J., Hall B., Graf N. et al. (2012) Human cytomegalovirus-induces cytokine changes in the placenta with implications for adverse pregnancy outcomes. PloS One 7, e52899 10.1371/journal.pone.0052899 23300810 PMC3534118

[190] Bergamelli M., Martin H., Benard M., Ausseil J., Mansuy J.M., Hurbain I. et al. (2021) Human cytomegalovirus infection changes the pattern of surface markers of small extracellular vesicles isolated from first trimester placental long-term histocultures. Front. Cell Dev. Biol. 9, 689122 10.3389/fcell.2021.689122 34568315 PMC8461063

[191] Tabata T., Petitt M., Li J., Chi X., Chen W., Yurgelonis I. et al. (2022) Neutralizing antibodies to human cytomegalovirus recombinant proteins reduce infection in an *ex vivo* model of developing human placentas. Vaccines (Basel) 10, 1074 10.3390/vaccines10071074 35891239 PMC9315547

[192] Coste Mazeau P., Jacquet C., Muller C., Courant M., El Hamel C., Chianea T. et al. (2022) Potential of anti-CMV immunoglobulin cytotect CP((R)) *in vitro* and *ex vivo* in a first-trimester placenta model. Microorganisms 10, 694 10.3390/microorganisms10040694 35456746 PMC9030298

[193] Tekkatte C., Lindsay S.A., Duggan E., Castro-Martinez A., Hakim A., Saldana I. et al. (2023) Identification of optimal conditions for human placental explant culture and extracellular vesicle release. iScience 26, 108046 10.1016/j.isci.2023.108046 37829201 PMC10565782

[194] Rosenthal L.J., Panitz P.J., Crutchfield D.B. and Chou J.Y. (1981) Cytomegalovirus replication in primary and passaged human placental cells. Intervirology 16, 168–175 10.1159/000149264 6277823

[195] Lu X., Wang R., Zhu C., Wang H., Lin H.Y., Gu Y. et al. (2017) Fine-tuned and cell-cycle-restricted expression of fusogenic protein syncytin-2 maintains functional placental syncytia. Cell. Rep. 21, 1150–1159 10.1016/j.celrep.2017.10.019 29091755

[196] Baczyk D., Drewlo S., Proctor L., Dunk C., Lye S. and Kingdom J. (2009) Glial cell missing-1 transcription factor is required for the differentiation of the human trophoblast. Cell Death Differ. 16, 719–727 10.1038/cdd.2009.1 19219068

[197] Alfaifi A.A., Heyder R.S., Bielski E.R., Almuqbil R.M., Kavdia M., Gerk P.M. et al. (2020) Megalin-targeting liposomes for placental drug delivery. J. Control. Release 324, 366–378 10.1016/j.jconrel.2020.05.033 32461116 PMC8247794

[198] Wu F., Tian F., Zeng W., Liu X., Fan J., Lin Y. et al. (2017) Role of peroxiredoxin2 downregulation in recurrent miscarriage through regulation of trophoblast proliferation and apoptosis. Cell Death Dis. 8, e2908 10.1038/cddis.2017.301 28661480 PMC5520946

[199] Halwachs-Baumann G., Weihrauch G., Gruber H.J., Desoye G. and Sinzger C. (2006) hCMV induced IL-6 release in trophoblast and trophoblast like cells. J. Clin. Virol. 37, 91–97 10.1016/j.jcv.2006.06.006 16884949

[200] Hyde K., Sultana N., Tran A.C., Bileckaja N., Donald C.L., Kohl A. et al. (2021) Limited replication of human cytomegalovirus in a trophoblast cell line. J. Gen. Virol. 102, 1683 10.1099/jgv.0.001683 PMC8742992 34816792

[201] Chou D., Ma Y., Zhang J., McGrath C. and Parry S. (2006) Cytomegalovirus infection of trophoblast cells elicits an inflammatory response: a possible mechanism of placental dysfunction. Am. J. Obstet. Gynecol. 194, 535–541 10.1016/j.ajog.2005.07.073 16458658

[202] Lin X., Chen Y., Fang Z., Chen Q., Chen L., Han Q. et al. (2020) Effects of cytomegalovirus infection on extravillous trophoblast cells invasion and immune function of NK cells at the maternal–fetal interface. J. Cell. Mol. Med. 24, 11170–11176 10.1111/jcmm.15638 32893994 PMC7576277

[203] Davis-Poynter N. and Farrell H.E. (2022) Constitutive signaling by the human cytomegalovirus G protein coupled receptor homologs US28 and UL33 enables trophoblast migration *in vitro*. Viruses 14, 391 10.3390/v14020391 35215985 PMC8879092

[204] Wang B., Fang Y., Wu Y., Koga K., Osuga Y., Lv S. et al. (2015) Viperin is induced following toll-like receptor 3 (TLR3) ligation and has a virus-responsive function in human trophoblast cells. Placenta 36, 667–673 10.1016/j.placenta.2015.03.002 25814471

[205] Kliman H.J., Nestler J.E., Sermasi E., Sanger J.M. and Strauss J.F. 3rd (1986) Purification, characterization, and* in vitro* differentiation of cytotrophoblasts from human term placentae. Endocrinology 118, 1567–1582 10.1210/endo-118-4-1567 3512258

[206] Li X., Li Z.H., Wang Y.X. and Liu T.H. (2023) A comprehensive review of human trophoblast fusion models: recent developments and challenges. Cell. Death Discov. 9, 372 10.1038/s41420-023-01670-0 37816723 PMC10564767

[207] Rollman T.B., Berkebile Z.W., Okae H., Bardwell V.J., Gearhart M.D. and Bierle C.J. (2024) Human trophoblast stem cells restrict human cytomegalovirus replication. J. Virol. 98, e0193523 10.1128/jvi.01935-23 38451085 PMC11019952

[208] Mimura N., Nagamatsu T., Morita K., Taguchi A., Toya T., Kumasawa K. et al. (2022) Suppression of human trophoblast syncytialization by human cytomegalovirus infection. Placenta 117, 200–208 10.1016/j.placenta.2021.12.011 34933151

[209] Okae H., Toh H., Sato T., Hiura H., Takahashi S., Shirane K. et al. (2018) Derivation of human trophoblast stem cells. Cell. Stem Cell. 22, 50.e56–63.e56 10.1016/j.stem.2017.11.004 29249463

[210] Haider S., Meinhardt G., Saleh L., Kunihs V., Gamperl M., Kaindl U. et al. (2018) Self-Renewing trophoblast organoids recapitulate the developmental program of the early human placenta. Stem Cell Rep. 11, 537–551 10.1016/j.stemcr.2018.07.004 30078556 PMC6092984

[211] Turco M.Y., Gardner L., Kay R.G., Hamilton R.S., Prater M., Hollinshead M.S. et al. (2018) Trophoblast organoids as a model for maternal–fetal interactions during human placentation. Nature 564, 263–267 10.1038/s41586-018-0753-3 30487605 PMC7220805

[212] Yang L., Liang P., Yang H. and Coyne C.B. (2024) Trophoblast organoids with physiological polarity model placental structure and function. J. Cell Sci. 137, jcs261528 10.1242/jcs.261528 37676312 PMC10499031

[213] Liu X., Wang G., Huang H., Lv X., Si Y., Bai L. et al. (2023) Exploring maternal–fetal interface with *in vitro* placental and trophoblastic models. Front. Cell Dev. Biol. 11, 1279227 10.3389/fcell.2023.1279227 38033854 PMC10682727

[214] Genbacev O., Donne M., Kapidzic M., Gormley M., Lamb J., Gilmore J. et al. (2011) Establishment of human trophoblast progenitor cell lines from the chorion. Stem. Cells 29, 1427–1436 10.1002/stem.686 21755573 PMC3345889

[215] Zydek M., Petitt M., Fang-Hoover J., Adler B., Kauvar L.M., Pereira L. et al. (2014) HCMV infection of human trophoblast progenitor cells of the placenta is neutralized by a human monoclonal antibody to glycoprotein B and not by antibodies to the pentamer complex. Viruses 6, 1346–1364 10.3390/v6031346 24651029 PMC3970154

[216] Tabata T., Petitt M., Zydek M., Fang-Hoover J., Larocque N., Tsuge M. et al. (2015) Human cytomegalovirus infection interferes with the maintenance and differentiation of trophoblast progenitor cells of the human placenta. J. Virol. 89, 5134–5147 10.1128/JVI.03674-14 25741001 PMC4403461

[217] Marsh B., Zhou Y., Kapidzic M., Fisher S. and Blelloch R. (2022) Regionally distinct trophoblast regulate barrier function and invasion in the human placenta. eLife 11, e78829 10.7554/eLife.78829 35796428 PMC9323019

[218] Yang L., Semmes E.C., Ovies C., Megli C., Permar S., Gilner J.B. et al. (2022) Innate immune signaling in trophoblast and decidua organoids defines differential antiviral defenses at the maternal–fetal interface. eLife 11, e79794 10.7554/eLife.79794 35975985 PMC9470165

[219] Takebe T., Zhang B. and Radisic M. (2017) Synergistic engineering: organoids meet organs-on-a-chip. Cell Stem. Cell. 21, 297–300 10.1016/j.stem.2017.08.016 28886364

[220] Park S.E., Georgescu A. and Huh D. (2019) Organoids-on-a-chip. Science 364, 960–965 10.1126/science.aaw7894 31171693 PMC7764943

[221] Cherubini M., Erickson S. and Haase K. (2021) Modelling the human placental interface* in vitro*—a review. Micromachines (Basel) 12, 884 10.3390/mi12080884 34442506 PMC8398961

[222] Wang Y., Guo Y., Wang P., Liu J., Zhang X., Liu Q. et al. (2025) An engineered human placental organoid microphysiological system in a vascular niche to model viral infection. Commun. Biol. 8, 669 10.1038/s42003-025-08057-0 40287582 PMC12033323

[223] Appios A., Thomas J.R. and McGovern N. (2021) Isolation of first-trimester and full-term human placental hofbauer cells. Bio. Protoc. 11, e4044 10.21769/BioProtoc.4044 34250210 PMC8250382

[224] Guo C., Cai P., Jin L., Sha Q., Yu Q., Zhang W. et al. (2021) Single-cell profiling of the human decidual immune microenvironment in patients with recurrent pregnancy loss. Cell. Discov. 7, 1 10.1038/s41421-020-00236-z 33390590 PMC7779601

[225] Jowett G.M., Read E., Roberts L.B., Coman D., Vila Gonzalez M., Zabinski T. et al. (2022) Organoids capture tissue-specific innate lymphoid cell development in mice and humans. Cell. Rep. 40, 111281 10.1016/j.celrep.2022.111281 36044863 PMC9638027

[226] Paris R., Apter D., Boppana S., D'Aloia M., De Schrevel N., Delroisse J.M. et al. (2023) Incidence of cytomegalovirus primary and secondary infection in adolescent girls: results from a prospective study. J. Infect. Dis. 228, 1491–1495 10.1093/infdis/jiad182 37340664 PMC10681855

[227] Georgountzou A. and Papadopoulos N.G. (2017) Postnatal innate immune development: from birth to adulthood. Front. Immunol. 8, 957 10.3389/fimmu.2017.00957 28848557 PMC5554489

[228] Arav-Boger R. (2015) Strain variation and disease severity in congenital cytomegalovirus infection: in search of a viral marker. Infect. Dis. Clin. North. Am. 29, 401–414 10.1016/j.idc.2015.05.009 26154664 PMC4552582

[229] Venturini C. and Breuer J. (2025) Cytomegalovirus genetic diversity and evolution: insights into genotypes and their role in viral pathogenesis. Pathogens 14, 50 10.3390/pathogens14010050 39861011 PMC11768282

